# Tree of Life Based on Genome Context Networks

**DOI:** 10.1371/journal.pone.0003357

**Published:** 2008-10-09

**Authors:** Guohui Ding, Zhonghao Yu, Jing Zhao, Zhen Wang, Yun Li, Xiaobin Xing, Chuan Wang, Lei Liu, Yixue Li

**Affiliations:** 1 Bioinformatics Center, Key Lab of Systems Biology, Shanghai Institutes for Biological Sciences, Chinese Academy of Sciences, Shanghai, People's Republic of China; 2 Graduate School of the Chinese Academy of Sciences, Shanghai, People's Republic of China; 3 College of Life Science & Biotechnology, Shanghai Jiao Tong University, Shanghai, People's Republic of China; 4 Shanghai Center for Bioinformation Technology, Shanghai, People's Republic of China; University of Western Cape, South Africa

## Abstract

Efforts in phylogenomics have greatly improved our understanding of the backbone tree of life. However, due to the systematic error in sequence data, a sequence-based phylogenomic approach leads to well-resolved but statistically significant incongruence. Thus, independent test of current phylogenetic knowledge is required. Here, we have devised a distance-based strategy to reconstruct a highly resolved backbone tree of life, on the basis of the genome context networks of 195 fully sequenced representative species. Along with strongly supporting the monophylies of three superkingdoms and most taxonomic sub-divisions, the derived tree also suggests some intriguing results, such as high G+C gram positive origin of Bacteria, classification of *Symbiobacterium thermophilum* and *Alcanivorax borkumensis* in Firmicutes. Furthermore, simulation analyses indicate that addition of more gene relationships with high accuracy can greatly improve the resolution of the phylogenetic tree. Our results demonstrate the feasibility of the reconstruction of highly resolved phylogenetic tree with extensible gene networks across all three domains of life. This strategy also implies that the relationships between the genes (gene network) can define what kind of species it is.

## Introduction

A highly resolved tree of life is a useful tool for biologist to make inferences about the dynamic processes of biological phenomena and to present evolutionary explanations [1]. Even though the horizontal gene transfer (HGT) is challenging the concept of tree of life and suggests using ticket-like network to depict evolution [2], [3], the backbone of the tree of life is intact [4], revealing the prevailing trend in the evolution of genome-scale gene sets or species [5]. This intact backbone tree could be inferred from the whole genome information.

To construct a species tree rather than gene trees, several phylogenomic methods were developed (reviewed in [6]). However, due to the compositional bias in sequence and rate variation bias across lineages and within sites [6], [7], a sequence-based phylogenomic approach leads to well-resolved but statistically significant incongruence, and “questions that are not resolved by a kilobase of sequence are seldom resolved by a megabase” [8]. In addition, phylogenetic reconstruction methods in terms of rare genomic changes (RGC) are limited to the production of highly resolved phylogenetic trees. This limitation stems mainly from the difficulty of true identification of these “Hennigian” markers, insufficient usage of the genomic information and the absence of statistical evaluation [9]. Thus, more sophisticated strategies are required to reconstruct the backbone tree of life as well as to test it independently.

**Figure 1:**
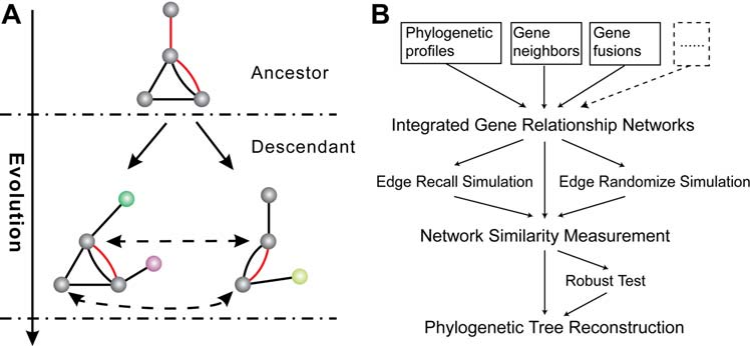
The principle and flowchart of the strategy. (A) The principle behind the evolution of gene relationship network and the alignment of the network. The balls denote different genes, while the colored edges denote different gene relationships, such as gene neighbor, co-expression [43] and so on. In evolution, the genes will be acquired (non-gray ball) or lost (some gray balls), and the same with relationships of genes. With the orthologous pairs, the extant gene networks in different organisms are aligned (dashed line with arrows). (B) The flowchart of the procedure. Firstly, integrate different gene relationships (e.g. phylogenetic profiles, gene neighbors and gene fusions in this work or more in the dashed box in the future) into multi-edge network. Secondly, align the networks based on the orthologous pairs and measure the similarity between every two networks to obtain a distance matrix. Thirdly, construct a phylogenetic tree based on distance matrix. Finally, validate the robustness of the derived tree and conduct simulations to understand the potential influence of accuracy and number of relationships of gene networks in our strategy.

As the question from the tale of the oracle at Delphi addressed, the relationships between the planks determine what kind of boat it is [10]. Similarly, in the evolution of the genomes, the relationships between the genes (gene networks), which make the genome function in their molecular and cellular contexts, determine what kind of species it is. Currently, with the development of computational methods for deriving gene networks from heterogeneous functional genomics data [11], [12] and measuring the similarity between two networks [13], it is possible to infer the tree of life from the comparison of gene networks among species. The guiding principle underlining this approach is that gene network is possibly the most subtle representation of the phenotype of an organism and vast amounts of evolutionary information may be hidden away within it (Figure 1A and Figure S1). In order to demonstrate the feasibility of this strategy, we have sought to construct a tree of life by considering the information contained within gene relationships at the genome level, as opposed to examining primary sequence identity. Such strategy have been tested on metabolic pathways [13], [14].

Herein we employed multi-edge gene-networks to represent the information of genomic gene relationships. These networks allow two or more edges linking the same gene-pair (Figure 1A) and associate evidence (e.g., the method to infer edges) as a property for each edge. We refer to such multi-edge gene-network as a “gene relationship network” (GRN). Ideally, if all the possible relationships among genes could be obtained, this network should be a full-information representation of an organism. Then, the difference between GRNs can be interpreted as a consequence of the fundamental properties of the species, which can be utilized to explore the tree of life. In practice, however, these differences can also be induced from the methods used to construct the networks. For example, more gene relationships can be found in model organisms than non-model organisms if using a literature mining method. Hence, in the absence of ideal GRNs, un-biased methods must be used to build the operational gene networks to approximate the ideal gene networks. In this work, we have used genome context networks (GCNs) in which nodes are referred as genes and edges can be inferred from genome context, as it is the only networks that could be constructed fairly for all genome-sequenced organisms now, to our knowledge.

## Results and Discussion

By integrating phylogenetic profiles, gene fusions and gene neighbors (Figure S2), we constructed GCNs from genome sequences of 195 organisms (Table S1). Then, pairwise comparison of GCNs was conducted to obtain a 195×195 distance matrix. With this matrix, we created a phylogeny of 195 species using the neighbor-joining algorithm [15]. To assess how strongly the data supports the resulting tree, a specific robustness test (see Material and Methods, Figure S3) corresponding to the traditional bootstrapping approach in phylogenetics was employed. The outline of this strategy is shown in Figure 1B.

### Tree Topologies

**Figure 2:**
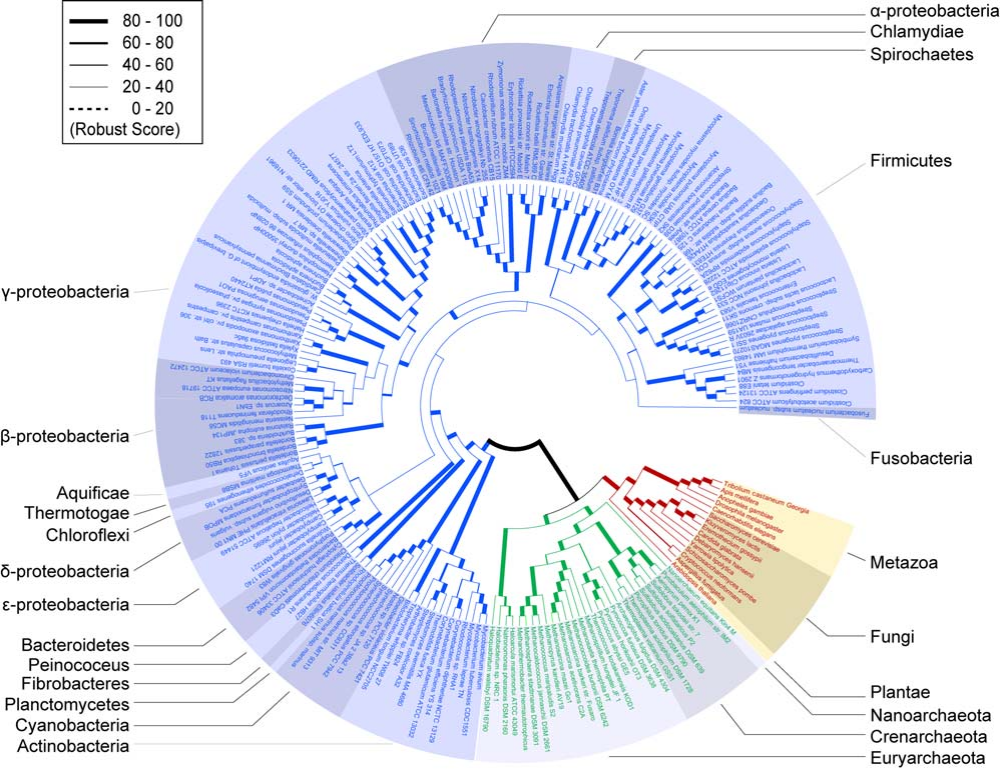
Tree of life based on genome context networks of 195 representative species. Robustness proportions are roughly represented by line width (see the legend at the upper left); exact numbers are given in Figure S4. Detailed discussion can be available in Table S4. Labels and color shadings denote various frequently used divisions. Red section, Eukaryota; green, Archaea; blue, Bacteria.

**Figure 3:**
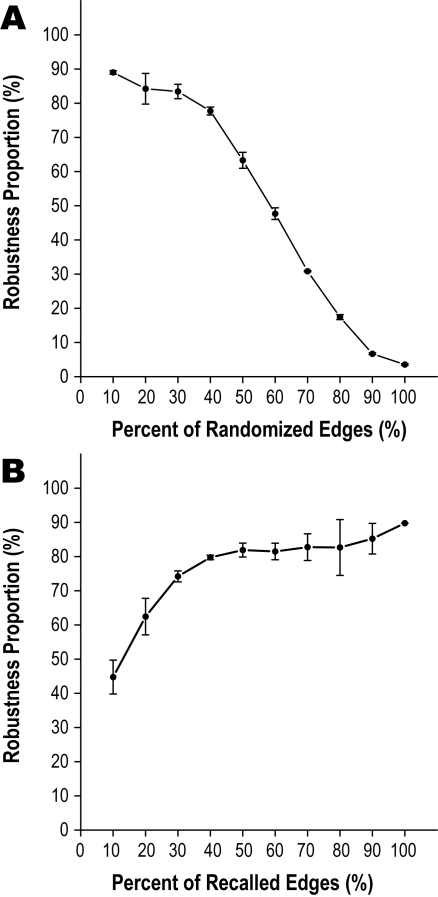
Influence of accuracy and number of edges in the gene networks on tree building. The template tree was based on the consensus tree in Figure 2 in both panels. The robustness proportions for template tree were constructed from 100 replicates of the networks generated in edge randomize simulation (up) or edge recall simulation (down). The data point and error bar represent the mean value of average robustness proportions in all template tree forks and plus/minus one standard error of 3 replicates. (A) Edge randomize simulation. The original gene networks are randomized by step of 10% from 10% to 100%. The result indicates that randomized gene relationship networks lead to unstable phylogenetic tree. (B) Edge recall simulation. The original gene networks are recalled by step of 10% from 10% to 100% too. The trend shown here indicates the growing amount of gene relationships or function information can improve the robustness of the resulting tree. With this result, the functional genomic research, such as protein-protein interaction analysis, can be helpful to resolve the universal tree of life.

Our strategy produces a highly resolved phylogenetic tree incorporating 195 species (Figure 2). Of all branches, 69.9% are supported by a robustness proportion (RP) of 100%, 80.3% with more than 80% RP support and 93.3% with more than 50% RP support. Because the resulting tree is inferred from gene relationships rather than primary sequence, it provides us an independent testing for our knowledge of the tree of life (Figure 2 and Table 1) and an opportunity to obtain deeper insight into the principle of the evolution of life. Consistent with previously constructed trees of life on the basis of combined protein sequences [16], [17] or sRNA [18], the tree from gene networks (Figure 2) strongly support the monophylies of the three domains (RP = 100%) and the close relationship between the Archaea and the Eukaryotes (RP = 100%) according to midpoint rooting. Within each domain, the monophylies of most major divisions can be confirmed (Table 1) and is well supported by high robustness values (RP>80%, see Figure 2 and the corresponding color shadings that indicate various divisions). The results of monophyletic divisions indicate that a specific gene network evolved in each taxonomic group, which can be used to distinguish one group from others. Interestingly, all weak RP divisions (RP<50%) are within the Bacterial domain toward the tips of our tree of life, but the deeper branches are all strongly supported (Figure 2). Hence, expanded data sets of gene relationships in the Bacteria could be used to further resolve the phylogeny of these weak RP divisions (Figure 3).

**Table 1:** Monophyly of each sub-division.

Domain	Sub-division	RP^a^ (%)
Eukaryota	Metazoa	100
	Fungi	100
	Plantae	-b
Archaea	Nanoarchaeota	-b
	Crenarchaeota	99
	Euryarchaeota (excluding Halobacteriaceae)	100
	Halobacteriaceae (a family of Euryarchaeota)	100
Bacteria	Actinobacteria	100
	Cyanobacteria	100
	Planctomycetes	-b
	Firbrobacteres	-b
	Peinococeus	100
	Bacteroidetes	100
	ε-proteobacteria	100
	δ-proteobacteria	100
	Chloroflexi	-b
	Thermotogae	-b
	Aquificae	-b
	β-proteobacteria	64
	γ-proteobacteria	96
	α-proteobacteria	100
	Chlamydiae	100
	Spirochaetes	100
	Fusobacteria	-b
	Mollicutes (a family of Firmicutes)	100
	Firmicutes (excluding Mollicutes)	56

The monophylies of most major divisions (9 out of 13 phyla) can be confirmed by high RP supports (RP>95%), implying the intrinsic differences in the gene networks of taxonomic divisions. The disruptions were found in Metazoa, Euryarchaeota, Proteobacteria and Firmicutes, even though the families in these phyla are monophyletic (RP>50%). Increased gene relationships in these genomes will help to resolve the phylogeny of these species (Figure 3). For detailed descriptions, see Table S4.

a RP stands for “Robustness Proportion”.

b Only one species of the phylum was used in our study.

### Bacterial Branch

**Figure 4:**
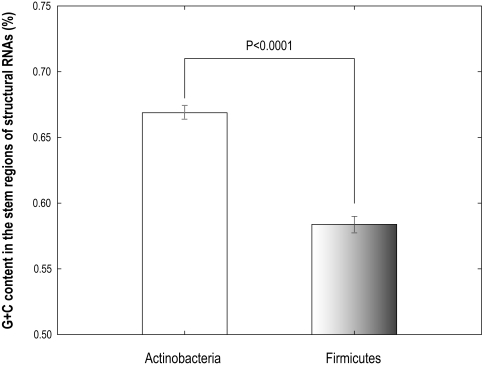
Comparison of G+C content in the stem regions of structural RNAs in Actinobacteria and Firmicutes. In this figure, data represent mean±standard deviation of G+C content in the stem regions from species in these two phyla respectively. The result of the comparison shows that species from Actinobacteria has a higher G+C content in the double-stranded stem regions of structural RNAs (mean = 0.669, standard deviation = 0.044) than those from Firmicutes (mean = 0.583, standard deviation = 0.063).

Our phylogenetic tree firmly places Actinobacteria (high G+C gram positive Bacteria) as the first bacterial branch (RP = 100%). This result is particularly intriguing, because it supports the theory of a gram positive origin of Bacteria [19] but proposes high G+C Bacteria (Actinobacteria) rather than low G+C ones (Firmicutes) in previous work [17], [20]. On average, the G+C content in the double-stranded stem regions of structural RNAs (tRNAs, 5S, 16S and 23S rRNAs) of Actinobacteria is significantly higher than that of Firmicutes (*p*<0.0001, un-paired t test; Figure 4), leaning to support the hypothesis of a thermophilic life-style of the common ancestor of Bacteria [18], [21], since high G+C content in structural RNA is necessary for survival in hot conditions [22], [23]. In addition, Actinobacteria is known to be particularly well adapted to survive in harsh environments (e.g. heavy metal-contaminated, deep sea, soil and so on) and thereby we could reason out the cruel living environment for original life on the earth.

In our consensus tree, the monophyletic photosynthetic Bacteria of Cyanobacteria are placed at the deep branch of the Bacteria after Actinobacteria (RP = 100%), indicating an early occurrence of oxygenic photosynthesis which is an important result for both biology and geochemistry [24]. Given credible fossil data for calibration, it is theoretically possible to date the age for Cyanobacteria based on our tree [25].

Within the clade of Firmicutes, the classification of *Alcanivorax borkumensis (SK2)* and *Symbiobacterium thermophilum (IAM 14863)* challenge the traditional taxonomy of these two species. The statistical support for assignment of *Alcanivorax borkumensis* in Firmicutes is strong (RP = 83%), whereas 16S rDNA tree placed it among γ-proteobacteria [26]. In contrast, its closely related species *Hahella chejuensis* in the 16S rDNA tree [26] clearly belongs to γ-proteobacteria in our result (RP = 100%). Therefore, the classification of *Alcanivorax borkumensis* should be reconsidered and we suggest that it is a species belonging to Firmicutes. For *Symbiobacterium thermophilum*, we grouped it with Clostridia (a class of Firmicutes) supported by a high RP value (RP = 100%) and then placed this cluster as a sister group to Bacilli (a class of Firmicutes; RP = 56%), in agreement with the proposal of sharing a common ancestor with Bacilli/Clostridia [27]. The previous classification of *Symbiobacterium thermophilum* into Actinobacteria on the basis of high G+C content and 16S rDNA [28] may be an artefact of G+C content bias [7]. Thereby, compositional characteristics according to primary sequence, such as G+C content, may not be sufficient for classifying an organism in taxonomy [27] and disturb the classification based on 16S rDNA [7]. Even though the *Symbiobacterium thermophilum* was assigned to Firmicutes, no more instances of Actinobacteria belonging to Firmicutes were detected here and vice versa [27]. In our tree, Actinobacteria and Firmicutes are two distinct clades, implying an intrinsic difference exists between the species in these two phyla at the gene networks level.

The relationships among Proteobacteria, Firmicutes, Bacteroidetes, Fusobacteria, Chloroflexi, Thermotogae, Aquificae, Chlamydiae and Spirochaetes are not well resolved (RP<50%), even though the monophyly is well supported in each sub-division (Table 1). The poor resolution of these clades may result from the amount of gene relationships used here (Figure 3) or taxon sampling [6]. Surprisingly, Thermotogae, Chloroflexi and Aquificae are grouped together too, albeit with weaker statistical support (RP<40%). Considering no point mutation information used in our strategy, the grouping of Thermotoga and Aquifex can't be explained as the result of the compositional bias of primary sequences [29] and thus puts forward the question of correlation of the core relationships of gene networks and the life-styles [30].

The artifactual clustering of Chlamydiae, Spirochaetes and Mollicutes (Figure 2, RP = 51%) could result from the parasitic nature of these species. 16 out of 17 genomes in these three classes were the ones with smallest genome context network sizes in our dataset (Table S1), in consistent with reductive evolutionary processes acting on the genomes of parasites [31]. The genome degradation process puts these species in a single statistical group, which was also found by the comparison of biochemical reaction pathways of 43 organisms [32]. Although the genome context network size of Spirochete *Treponema denticola* is two times the sizes of other species in Spirochaetes (Table S1), all these species in Spirochaetes were clustered together. And *Nanoarchaeum equitans Kin4-M* with the smallest network size is grouped in Archaea, as expected. Furthermore, the other parasites with the larger genome context network sizes are posited in the expected phyla. Therefore, the phylogenetic signatures in the parasites genome context networks are sufficient to classify the organism in domain level and support the monophylies of these sub-divisions.

### Archaeal and Eukaryal Branch

In Archaea, the deepest branch is Halobacteriaceae, which is one family of Euryarchaeota. It is placed as a closer sister group to the cluster consisting of Crenarchaeota, Nanoarchaeota and other Euryarchaeota families (RP = 100%). Another result in Archaea is that the Nanoarchaeota emerged before the split of Euryarchaeota (excluding family of Halobacteriaceae) and Crenarchaeota and after Halobacteriaceae (RP = 99%), which further fuels the controversy regarding the position of Nanoarchaeota [17], [33]. These results suggest that additional sequenced Archaeal genomes will help to decipher the history of the Archaeal superkingdom.

In Eukaryota, with the exception of Deuterostomia, the branching orders agree with current evolutionary knowledge (Figure S6) [6]. That Deuterostomia (Table S2) is placed in the deep branch after Plantae before Fungi (Figure S6) is probably due to “big networks attraction” (Text S1 and Figure S5) similar to big genome attraction [34], and large numbers of paralogous genes from whole genome duplications in ancestral Vertebrate [35] that hamper the assignment of orthologous pairs in terms of protein sequences. Further studies on the processes of Eukaryotic genome and gene network evolution (e.g., more realistic mathematical models) are required to clarify their high order systematics in terms of gene networks.

### Features of the strategy

Compared to the traditional phylogenomic approaches based on primary sequence, our strategy only makes use of gene relationships so as to be immune to system errors caused by compositional bias, within-site rate variation and so on. On the other hand, in contrast to classical methods in terms of rare genomic changes [9], we examined the comprehensive relationships of genes in whole genome which encompass these rare genomic changes. Because of the difficulty in identifying the rare genomic changes [9], the phylogenetic signals of a few RGCs are insufficient to resolve phylogenetic tree [9], whereas our strategy can obtain a highly resolved tree of life (Figure 2 and Table 1). Another important point here is that the horizontal gene transfer (HGT) has little influence on building a phylogenetic tree based on gene networks. Previously, many phylogenetic anomalies were simply explained as dilution of phylogenetic signal by HGT [17]. However, while there are many factors that could generate such discrepancies in phylogenetic reconstruction [36], e.g., biased mutation rates on the primary sequences, our approach is not affected by these factors. In addition, if a gene is laterally transferred into one genome from another, it will have little impact on the essential function of an organism [36], i.e., with few relationships to other genes from vertical evolution, resulting in smaller network-structural similarities of these genes. Furthermore, it is a small probability event that the relationship of two genes in vertical evolution would be maintained when a gene was a laterally transferred gene, such as the relationship of gene positions [37], [38], even though it might theoretically occur. If the alien genes persisted within the host genome very long, they were possible to be fully integrated in the host genome networks to destroy the assumption of smaller network-structural similarities of these genes. However, alien genes tend to be purified by selection and to be transient residents in the host genome due to the natural barriers to oppose the invasiveness of transferred sequences [36], [39]. Accordingly, the putative laterally transferred genes have little contribution to the distance calculation in our method.

The distance used to construct the phylogeny in our strategy is a measure of the conservation of gene relationships between two organisms, which is completely different from the gene content method that is based on the conservation of shared genes (Figure S1, Table S5). The main contribution of the distance is the relationships of gene in the genome context. If the genes were independent of others, our method will give similar result as the gene content methods. However, the relationships between genes should be considered for the actual organisms [40] and be used for phylogenetical analysis.

To better understand the influence of accuracy and number of edges in the networks on the resolution of phylogenetic tree and to explore the trends when more data are available, we conducted simulation analyses on the consensus tree in Figure 2 (see Material and Methods). The level of statistical support, which was expressed as the mean value of robustness proportion for internal nodes observed in the template tree, was plotted as a function of the factors to be simulated (Figure 3). With a step of 10% to randomize the network, we performed an edge randomize simulation to study the effect of the randomized edges in the gene network, showing a significant negative correlation between the statistical support and randomizing degree of the network (*p*<0.0001; r = −0.9790; Figure 3A). As shown in Figure 3A, the statistical support drops down slowly when the randomizing degree is less than 40%, indicating the error-tolerance of our approach. It decreases rapidly with increasing randomizing degree greater than 40%, suggesting that a mass of error edges in the gene networks can produce an unstable phylogenetic tree. To simulate the effect of increasing the number of edges, we conducted an edge recall simulation with a step size of 10% to recall the entire gene network. As seen in Figure 3B, the statistical support correlates positively with the proportion of the recalled edges in the whole gene network (*p*<0.01; r = 0.8458; Figure 3B). Similarly, a critical point of 40% is found in Figure 3B. When the proportion of recalled edges is greater than this value, the statistical support rises slowly, which hints that phylogenetic signals in more than 40% of the ideal gene relationship data are sufficient to generate a highly resolved phylogenetic tree (RP>80%). The results of these simulations suggest that adding more edges with high accuracy in the gene network will greatly increase the resolution of the phylogenetic tree.

In essence, the GCN is a mathematical abstraction of the macrostructure of the entire genome. However, it makes sense biologically. The GCNs here are gene function linkage networks [11], [12], [40], [41]. What is more, the emergence of a more complex and integrated network of genes is a key transition in the evolution of Darwinian lineages [42], i.e., the gene network or the connectivity of the genes is the basis for Darwinian evolution. Thereby, GCN is ideal for describing gene relationships of an organism on genome level, and can be used to reconstruct a phylogenetic tree. Nonetheless, the GCN is only part of the ideal gene relationship network, and some other gene relationship data that are illustrated as dashed box in Figure 1B, such as protein-protein interaction data, co-expression data [43], can be integrated in the future or analysed solely.

### Conclusions

We have presented a tree of life based on GCNs and demonstrated the feasibility, potential and trends of this strategy. The derived tree here sheds new light on the evolutionary history of organisms and their genomes, by retrieving and comparing their GRNs that define what kind of organisms they are. In addition to challenging some traditional taxonomies, the tree also provides new view for studies on relationships between organisms and their living environment and serves as a background taxonomy for meta-genomics. Our strategy emphasizes that a gene should be defined as an element in the network of its interactions, in agreement with the post-genomic view of gene function [40]. Beyond sequencing more species, the research on gene function or relationships is valuable for further resolving the universal tree of life, as well as further understanding the evolution of the organisms on the gene networks level.

## Materials and Methods

### Data sets collection

More than 1400 genome projects are recorded in the NCBI Entrez Genome Project database (Archaea with 62 projects, Eukaryotes with 438 projects and Bacteria with 1086 projects can be browsed in February 24, 2007). But considering the computational load and feasibility, 195 representative species were chosen (Table S1) and the sequence data with corresponding annotation information were downloaded from the ftp of NCBI RefSeq Project (ftp://ftp.ncbi.nih.gov/genomes/, accessed Oct., 2006). Deuterostomia (Table S2) were excluded due to big networks attraction (Table S4) and lots of paralogous genes from whole genome duplications in ancestral Vertebrate [35], which is detailedly discussed in the Text S1. Five sub-strains of Escherichia coli were added to the data sets because of broad scientific interests in this species and testing of the resolution of sub-strains in the same species in our strategy. According to the endosymbiosis hypothesis for the origin of Mitochondria and Chlorroplasts [18], genes deposited in these two organelles were taken out from the Eukaryotes. Similarly, plasmid genes were taken out from Bacteria and Archaea genomes. This is because, being not the core of the organisms, these genes express some assistant function, and tend to horizontal transfer. The NCBI GI number list of protein sequences after pretreatment can be downloaded from the website associated with this work (http://www.biosino.org/papers/gcnEvol).

### Identification of homologous genes

All-to-all protein sequence similarity search from collected dataset was performed using gapped BLASTP (version 2.2.10) [44] with default setting. Low complexity sequences were filtered with SEG [45], and 10^−5^ was chosen as the E value cut-off. Two genes were identified as homologous genes if and only if the longest protein sequences encoded by these two genes satisfy all of the four criteria: (i) all High-scoring Segment Pairs (HSPs) are compatible with the global HSPs arrangement on the protein sequence, or else to remove it [46]; (ii) the remaining HSPs cover more than 70% of the protein length; (iii) the similarity of each HSPs is more than 50% (two amino acids are considered similar if their BLOSUM62 similarity score is positive) [46]; (iv) these conditions are symmetrical for both genes. We used the smallest E value of the HSPs from two genes as an index to define the putative orthologous gene pair, i.e., the putative orthologous gene of gene A is the gene in corresponding organism with the smallest E value index of all homologous genes for gene A. That is an operational definition of “orthologous gene” (more discussion in the Text S1), whose independent evolution reflects a speciation event.

### Construction of genome context networks

Phylogenetic profiles method, gene neighbors method and gene fusions method were adopted to construct the genome context networks [41]. In Phylogenic profiles method, pairs of proteins with similar patterns of presence and absence across genomes were identified. In gene neighbors and fusion method, pairs of genes that fused or clustered together during evolution were detected. All gene pairs were set a *p* value in contrast to random situation. Then, a threshold of *p* value was used to select the gene relationship with small *p* value (Table S3). With these gene relationships, a multi-edge network, which allows more than one edge linking the same node pair, will be constructed. For details, see Protocol S1, Figure S2 and Table S3.

### Distance measurement for network-pairs

To reduce the computational complexity, the network-pair is pre-aligned by strict ortholologous pairs identified according to sequence information. This simplified strategy is consistent with the naive approach, as the object of the naive approach is that the orthologous genes in two networks from two different organisms should be aligned correctly. Afterward, we defined gene similarity in the networks by Jaccard index. For highlighting the gene relationships, the similarity or distance of the primary protein sequence was neglected and only the structural similarity between orthologous genes was used. This similarity of genes on network level is given as:
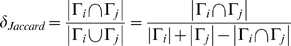
(1)where *Г_i_(Г_j_)* is the set of edges (relationships) linking to gene *i*(*j*) and |*Г*| denotes the set size of *Г*. The common edges (relationships) are the edges with orthologous neighborhood and same inferring method. Finally, the similarity score of two gene-networks was calculated by summing all similiarity scores calculated over pair of orthologous genes and normalized the sum by the square root of the product of the genes in these two gene-networks, which can be formulated as:
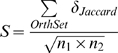
(2)where *OrthSet* is the set of orthologous pairs in these two organisms, *δ_Jaccard_* is the Jaccard index of the orthologous genes on the network level defined in Eq. 4, *n*_1_ and *n*_2_ are the numbers of the genes in these two gene networks [13]. When the similarity score was obtained, the distance can be computed by the formula of *d = *1*−S*.

### Phylogenetic tree inference and robustness test

As the distance measurement of two gene networks from organisms was defined, a distance matrix can be obtained by comparing each species against all the others. Based on this distance matrix, neighbor-joining method [15] was applied to construct the phylogenetic tree, which was implemented by the program neighbor in PHYLIP package [47].

To valudate the robustness of the resulting tree inferred from genome context networks, a specially designed robustness test (Figure S3) similar to traditional bootstrapping approach used in phylogenetics was utilized. The original list of orthologous gene-pairs with Jaccard index of two networks is uniformly re-sampled with replacement to produce pseudo-replicate data sets (Figure S3). The similarity score between these simulated networks was then calculated by summing these indexes in the obtained list of orthologous gene-pairs, and then normalizing and transforming to distance by the methods used in original data. This process was repeated *m* times (*m* = 100 in our study). A set of distance matrices was generated, and then was used for building *m* phylogenetic trees by neighbor-joining method [15]. The consensus tree can be inferred from these simulated trees, e.g., Figure 2. A robustness statistic of a fork, named robustness proportion (RP), was applied to indicate how many times a group which consists of the species to the right of (descended from) the fork occurred in the generated tree set. Accordingly, the mean value of these robustness statistics of all forks in a phylogenetic tree suggests the robustness of this tree.

### Simulation analyses

In order to better understand the influence of accuracy and number of edges in the networks on building phylogenetic tree and to explore the trends when more data are available, simulation analyses were conducted. The template tree was based on the genome context networks of 195 species, as shown in Figure 2. Based on this template tree, the accuracy and the number of the edges were changed in a stepwise style and the mean value of the RPs in all forks was regarded as dependent variable (Figure 3). In the edge randomize simulation, we randomized the proportion of the edges in the networks with a step of 10% from 10% to 100% to produce pseudo-replicate networks. In the edge recall simulation, the addition of edges in the networks was also with a step of 10% from 10% to 100%. At each step of both simulations, the generated networks were applied for tree building and a mean RP value was obtained. We repeated this process 3 times and estimated the variance of the simulations (error bar in the Figure 3). Note that, small number of replicates (3 times) was due to the huge computational resource required for each process in simulation.

### Comparison of G+C content in the double-stranded stem regions of structural RNAs in Actinobacteria and Firmicutes

The tRNAs, 5S, 16S and 23S rRNAs sequences were used as structural RNAs herein. We predicted the secondary structures of these RNAs with Afold [48] which are available at the supporting website (http://www.biosino.org/papers/gcnEvol). Sequences in the double-stranded stem regions of the RNA structures were extracted to calculate the G+C percentage. In addition, we applied RNAfold [49] to predict RNA secondary structures and conducted the same analysis. Same result was obtained (data not shown).

## Supporting Information

Protocol S1 (0.03 MB DOC)

Text S1 (0.05 MB DOC)

Figure S1 Illustration of Gene Content based Method and Gene Network based Method.
(0.20 MB PDF)

Figure S2 Rationales of three methods to construct the genome context networks in this work.
(0.09 MB PDF)

Figure S3 Illustration of the robustness test in the gene network alignment with bootstrapping approach.
(0.17 MB PDF)

Figure S4 Rectangular cladogram of phylogenetic tree of 195 representative species.
(0.15 MB PDF)

Figure S5 Distribution of genome context work sizes.
(0.09 MB PDF)

Figure S6 Phylogenetic tree based on genome context network after addition of Deuterostomia.
(0.15 MB PDF)

Table S1 Species used in this work.
(0.27 MB DOC)

Table S2 Species of Deuterostomia used in the big network attraction experiment.
(0.04 MB DOC)

Table S3 Edge numbers in genome context networks.
(0.03 MB DOC)

Table S4 Evidence to support the monophyly of each sub-division.
(0.09 MB DOC)

Table S5 Comparison of Gene Content/Networks Based methods in methodology
(0.27 MB PDF)
