# Supplementary material for: Tree of Life Based on Genome Context Networks
Source: PLoS One. 2008 Oct 9;3(10):e3357. doi: 10.1371/journal.pone.0003357 (PMC2566592; doi:10.1371/journal.pone.0003357)
Supplement: Protocol S1 — (0.03 MB DOC) [file pone.0003357.s001.doc]

### Supporting Protocol 1

**Construction of genome context networks**

Three methods including phylogenetic profiles method, gene neighbors method and gene fusions method were adopted to construct the genome context networks. These methods are very popular in predicting protein function linkages and are all well discussed and performed [1,2]. Therefore, we just describe the rationales briefly (Supplemental Fig. 1). The orthologous information used in these methods was derived from the methods mentioned in the text.

In Phylogenic profiles method (Supplemental Fig. 1A) [1], we identified pairs of proteins with similar patterns of presence and absence across genomes. Firstly, every protein was given an *N*-dimensional vector (*N* equals the total number of organisms used) composed of 1s and 0s denoting the presence (with 1) and absence (with 0) of an orthologous gene of the query protein in the corresponding genome. Then the *p*-value of each protein pair can be calculated by using the hypergeometric distribution

$P(x=k|N,m,n)=\frac{\left(\begin{matrix} n \\ k \end{matrix}\right)\left(\begin{matrix} N-n \\ m-k \end{matrix}\right)}{\left(\begin{matrix} N \\ m \end{matrix}\right)}$ (1)

where *N* is the total number of genomes, *n* the number of orthologs for protein A, *m* the number of orthologs for protein B and *k* the number of genomes that contain orthologs of both A and B. By setting different *p*-value cutoffs, we can generate linkage pairs of proteins or pairs of genes by mapping the proteins to gene loci (Table S3).

In gene neighbors method (Fig. 1B) [1], we can detect genes that are clustered together. Firstly, compute the probability that two genes are separated by fewer than *d* genes using the location information of each gene:

$P(x\leq d)=\frac{2d}{N-1}$ (2)

where *N* is the total number of genes on the genome. Then by calculating the geometric mean of the product of probability across all organisms the two genes both has its orthologous gene, we got the *p*-value for evaluating the neighbor relationship of two genes based on this method.

In gene fusions method (also known as the Rosetta Stone method; Supplemental Fig. 1C) [1], we detected pairs of genes that fused together during evolution. Pairs of non-homologous proteins both align over 70% of their sequence to a third protein were recorded and the hypergeometric distribution was used to generate the *p*-value too:

$P(x=k|N,m,n)=\frac{\left(\begin{matrix} n \\ k \end{matrix}\right)\left(\begin{matrix} N-n \\ m-k \end{matrix}\right)}{\left(\begin{matrix} N \\ m \end{matrix}\right)}$ (3)

where *k* is the number of Rosetta Stone sequences, *n* the number of orthologs of protein A and *m* the number of orthologs of protein B and *N* the total number of sequences.

1. Bowers PM, Pellegrini M, Thompson MJ, Fierro J, Yeates TO, et al. (2004) Prolinks: a database of protein functional linkages derived from coevolution. Genome Biol 5: R35.

2. Eisenberg D, Marcotte EM, Xenarios I, Yeates TO (2000) Protein function in the post-genomic era. Nature 405: 823-826.
