# Supplementary material for: Tree of Life Based on Genome Context Networks
Source: PLoS One. 2008 Oct 9;3(10):e3357. doi: 10.1371/journal.pone.0003357 (PMC2566592; doi:10.1371/journal.pone.0003357)
Supplement: Text S1 — (0.05 MB DOC) [file pone.0003357.s002.doc]

### Supporting Text 1

**The operational definition of “orthologous gene”**

The criteria to determine the orthologous genes were based on the HOBACGEN[1] and InParanoid [2]. To decrease the computational load, we employed the best bidirectional hit (BBH) method to detect orthologous genes. Even though more sophisticated method has been developed to find orthologous genes [3], the biases in these methods are evident [4]. Thus, we applied the direct and simple method to define ortholgous genes.

In order to study the effect of the criteria to detect the orthologous gene on the resulting tree, we have tried the strict and loose criteria through the threshold of the overlap of the protein sequence (from 60% to 90% by a step of 10%). The threshold of 80% is a critical point. When we used the strict criteria with overlap ≥ 80%, the phylogenetic signatures are not sufficient to construct a resulting tree. While the overlap < 80%, the topology of the resulting tree is stable. In the following analysis, we made use of a moderate threshold of 70%. However, the attraction of Deuterostomia gene networks, which will be discussed in the following text, can not be solved even if we changed the criteria.

We also tried the orthologous pairs in the COG database [5]. As the species in the COG database are limited (66 genomes, August, 2007), we randomly chose a small subset out of the intersection of our species set and that of COG database (18 organisms in this study). The topologies of the resulting trees based on tested species are the same.

**Selection of *p* value of the gene relationship has less impact on the result**

The *p* values of 0.01 and 0.1 were also used as the cutoffs of relationship (Table S3) to construct the genome context networks. Based on these networks, the derived phylogenetic trees are almost the same as that from networks filtered with *p* value of 0.05 in the text (data not shown). The main conclusions, such as tripartite scheme of life-forms (Eukaryota, Bacteria, Archaea), high G+C gram positive origin of bacteria, monophylies of most taxonomic divisions, were all strongly supported (RP > 80%).

**Contribution of three network-building methods in the construction of the phylogenetic tree**

The networks built from individual method were used to construct the phylogenetic tree and compared with the consensus tree based on full data. As expected in the edge recall simulation, fewer edges from sole method result in less robustness of the phylogeny tree. In this study, only the gene relationships from gene fusions method are not sufficient to construct the full tree of life (Table S3). Against to the template tree (the consensus tree in Fig. 2 in the text), the mean values of robustness proportion in all forks based on gene neighbors method and phylogenetic profiles method are 87.01036 and 43.96891 respectively, indicating the major contribution of the gene neighbors method. However, comparing the phylogeny based on gene order in previous work [6], the topology of our consensus tree is obviously different from it, e.g., the branch order of species in Bacteria. Therefore, the integrated data give us a more comprehensive insight into the evolution of organisms and their genomes. In the further study, more gene relationships, such as co-expression, protein-protein interaction data, should be added to construct the universal tree of life by this strategy.

**Big network attraction in the Deuterostomia genomes**

As shown in Supplemental Fig. 4, the mean size of Deuterostomia gene networks is larger than others with statistical significance (*p* < 0.05, nonparametric test). If the size of the networks are huger than normal networks, these networks trend to possess small similarities with others as the denominator in the Eq. 2 in the text will be large and to be clustered together. So we excluded the Deuterostomia genomes in our work. Extensive species sampling lower than Deuterostomia in evolutionary distance should be an approach to solve this problem, as additional network sizes buffer the sharply big networks compared to others. In addition, the development of more realistic modes of the processes of genome and gene network evolution may be helpful to further progress in resolving this problem.

However, we did an experiment to add 8 Deuterostomia genomes (Table S2). Expectably, the species of Deuterostomia were clustered together but placed at the deep branch before fungi and Endopterygota, which may be an artifact of big networks of these organisms (Supplemental Fig. 5). Howbeit other topological characteristics in this tree are similar to the consensus tree in Fig. 2 of the text (Supplemental Fig. 5). Further analyses are required to clarify the phylogenetic relationships among species in Eukaryota based on gene networks.

We also checked the gene network sizes among sister taxa in phylogenetic trees including 195 species or 203 species and no obvious correlations are found. For example, *Nanoarchaeum equitans Kin4-M* with the smallest networks (169 nodes) is adjacent to the group with average network size of 1186 (±471) nodes rather than another group with average network size of 1011 (±196) nodes in Archaea. Similar relationships are found in the other clades, as well. These founds imply that big network attraction did not appear to be significant for our dataset with normal network sizes (1786 ± 1318 nodes).

1. Perriere G, Duret L, Gouy M (2000) HOBACGEN: database system for comparative genomics in bacteria. Genome Res 10: 379-385.

2. O'Brien KP, Remm M, Sonnhammer EL (2005) Inparanoid: a comprehensive database of eukaryotic orthologs. Nucleic Acids Res 33: D476-480.

3. Hulsen T, Huynen MA, de Vlieg J, Groenen PM (2006) Benchmarking ortholog identification methods using functional genomics data. Genome Biol 7: R31.

4. Snel B, Bork P, Huynen MA (1999) Genome phylogeny based on gene content. Nat Genet 21: 108-110.

5. Tatusov RL, Fedorova ND, Jackson JD, Jacobs AR, Kiryutin B, et al. (2003) The COG database: an updated version includes eukaryotes. BMC Bioinformatics 4: 41.

6. Korbel JO, Snel B, Huynen MA, Bork P (2002) SHOT: a web server for the construction of genome phylogenies. Trends Genet 18: 158-162.
