# Supplementary material for: Tree of Life Based on Genome Context Networks
Source: PLoS One. 2008 Oct 9;3(10):e3357. doi: 10.1371/journal.pone.0003357 (PMC2566592; doi:10.1371/journal.pone.0003357)
Supplement: Figure S1 — Illustration of Gene Content based Method and Gene Network based Method. (0.20 MB PDF) [file pone.0003357.s003.pdf]

Figure S1. Illustration of Gene Content based Method and Gene Network based Method.

1. Snel, B., Bork, P. and Huynen, M.A. (1999) Genome phylogeny based on gene content. *Nat Genet*, **21**, 108-110.
2. Leicht, E.A., Holme, P. and Newman, M.E. (2006) Vertex similarity in networks. *Phys Rev E Stat Nonlin Soft Matter Phys*, **73**, 026120.

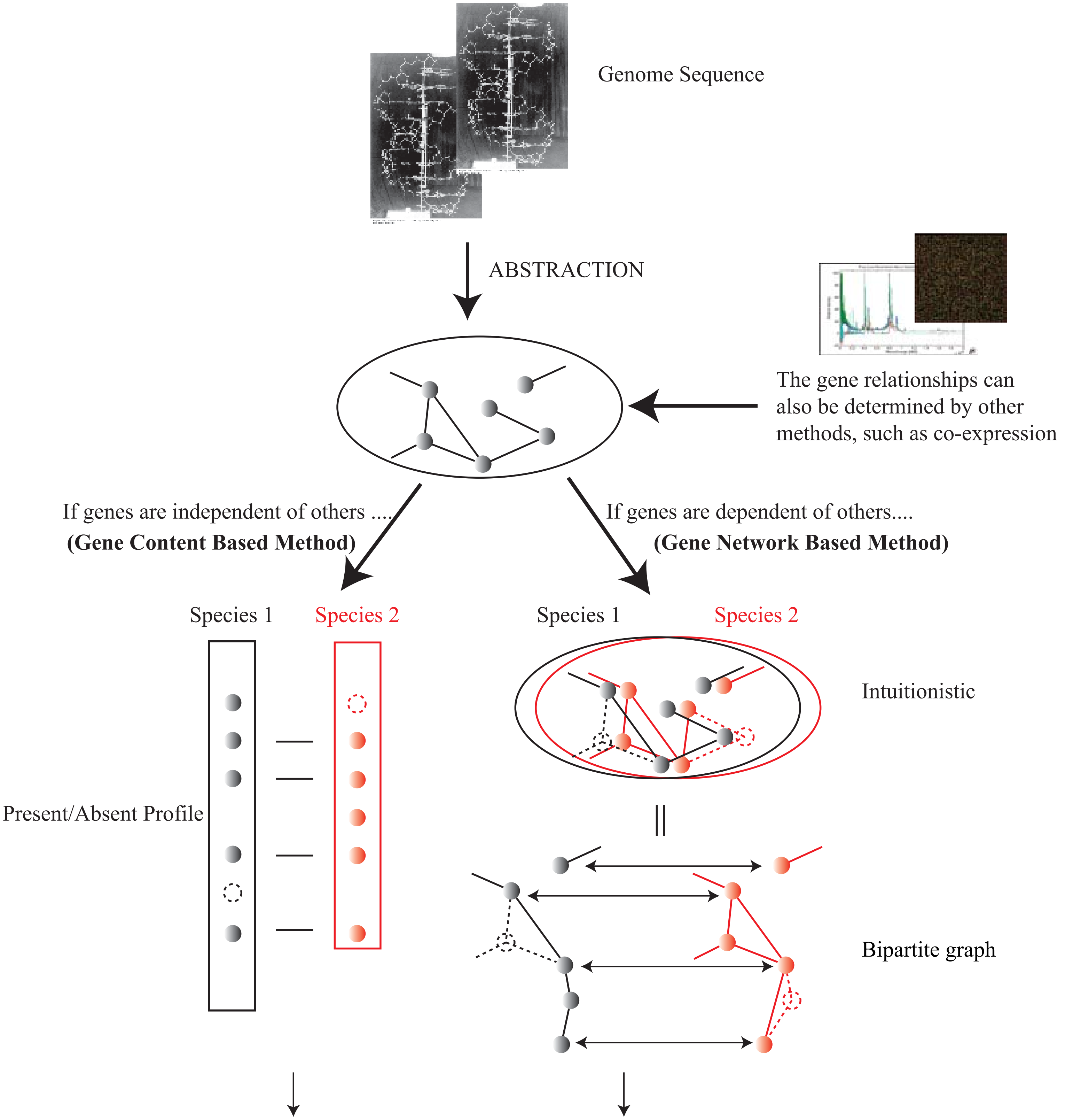

Distance Measure based on the number of shared genes, genes in species 1 and 2. For example, in Snel's paper [1], $d(G1, G2) = 1 - |G1 \cap G2| / \min(|G1|, |G2|)$

Distance Measure based on the scores of matched nodes. In network level, the score of two matched nodes is the product of structural similarity in network level (δ1) and the similarity of these two themselves (δ2). The structural similarity can be produced by the definition of structural equivalence or regular equivalence [2].When we set δ2 = 1, then the distance of two networks was only depend on the gene relationships in the network. If we assum the genes were independed, and all the structural partions of the scores are equal (given δ1 = δ2 = 1), we will get a result similar to gene content method.

Figure S1
