# Supplementary material for: Tree of Life Based on Genome Context Networks
Source: PLoS One. 2008 Oct 9;3(10):e3357. doi: 10.1371/journal.pone.0003357 (PMC2566592; doi:10.1371/journal.pone.0003357)
Supplement: Figure S2 — Rationales of three methods to construct the genome context networks in this work. (0.09 MB PDF) [file pone.0003357.s004.pdf]

Figure S2. Rationales of three methods to construct the genome context networks in this work.

Genomes are represented as line (A and C) or circle (B), and genes as rectangles (A and C) or circle (B) with different color. Genes with same color denote the orthologous relationships of these genes. This figure is modified from [1].

(A) Construction of phylogenetic profiles. A matrix of presence (with 1) and absence (with 0) is derived from four demo genomes and the column of this matrix is also named as "phylogenetic profile" of a gene. Then, it is easily to find that the 'orange' and 'yellow' genes have the same phylogenetic pattern among the four genes. By comparing different vecters, we can generate *p*-values of different pairs.

(B) Illustration of gene neighbors method. It identifies protein pairs encoded in close proximity across multiple genomes. Four genomes are illustrated here; among them, gene 'red' and gene 'yellow' are conservative gene neighbors while the others are not.

(C) Demonstration of gene fusions method. It was used to search gene fusion events which had been employed as phylogenetic signatures [2]. Seen from the demonstration, gene B and gene C are expressed separately in one genome but their orthologous genes are fused into one gene (gene A) in other genome or the same genome. Both B and C align over a certain part of their sequence against the third gene C in protein sequences.

1. Bowers PM, Pellegrini M, Thompson MJ, Fierro J, Yeates TO, et al. (2004) Prolinks: a database of protein functional linkages derived from coevolution. Genome Biol 5: R35.
2. Stechmann A, Cavalier-Smith T (2002) Rooting the eukaryote tree by using a derived gene fusion. Science 297: 89-91.

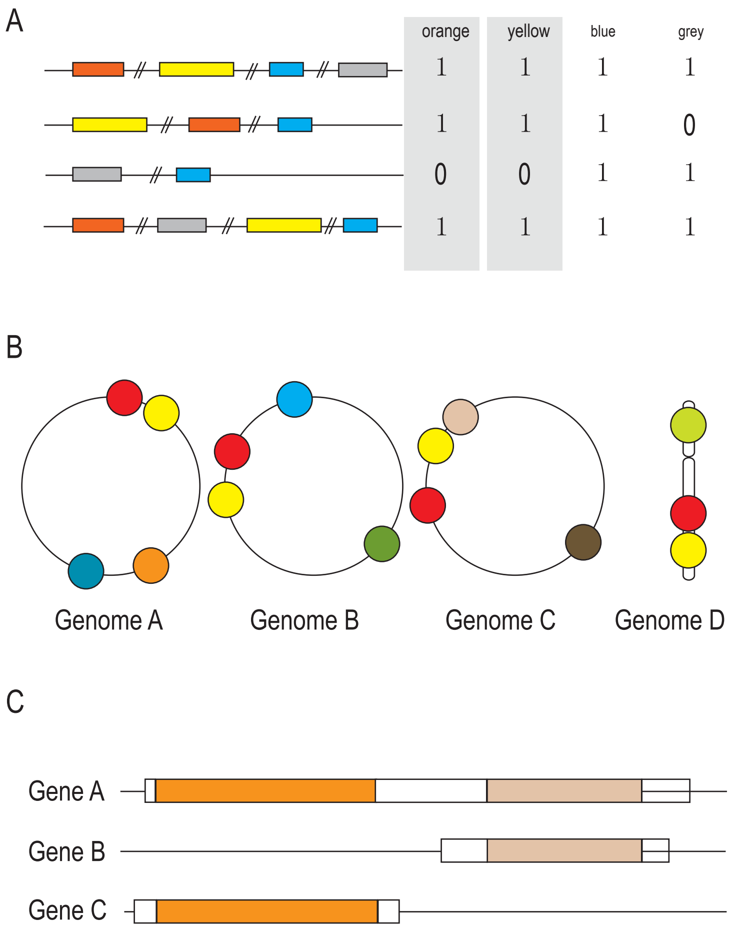
A
orange
yellow
blue
grey
1
1
1
1
1
1
1
0
0
0
1
1
1
1
1
1
B
Genome A
Genome B
Genome C
Genome D
C
Gene A
Gene B
Gene C

Figure S2
