# Supplementary material for: Tree of Life Based on Genome Context Networks
Source: PLoS One. 2008 Oct 9;3(10):e3357. doi: 10.1371/journal.pone.0003357 (PMC2566592; doi:10.1371/journal.pone.0003357)
Supplement: Figure S3 — Illustration of the robustness test in the gene network alignment with bootstrapping approach. (0.17 MB PDF) [file pone.0003357.s005.pdf]

Figure S3. Illustration of the robustness test in the gene network alignment with bootstrapping approach.

In the panel, gray balls denote the orthologous genes in two organisms (OP# or OP#' in the figure), while the colored balls represent the genes without orthologous counterparts in the other organism. By gray balls, two demo networks were pre-aligned, rendered as dashed lines with arrows. On this dashed arrow lines, similarity values were displayed (δ# in the figure and Jaccard index used in our study). Then, we regarded this alignment as a simply list with element of orthologous gene pair (original list). The bootstrapping method was applied on this original list. Here, the uniform distribution was used to resample the elements in the original list to generate artificial list. This process was repeated *m* times to produce a dataset with *m* pseudo-lists (sample list_# in the figure). All the procedures employed in the original list were applied on this dataset and we could obtain the robustness statistics.

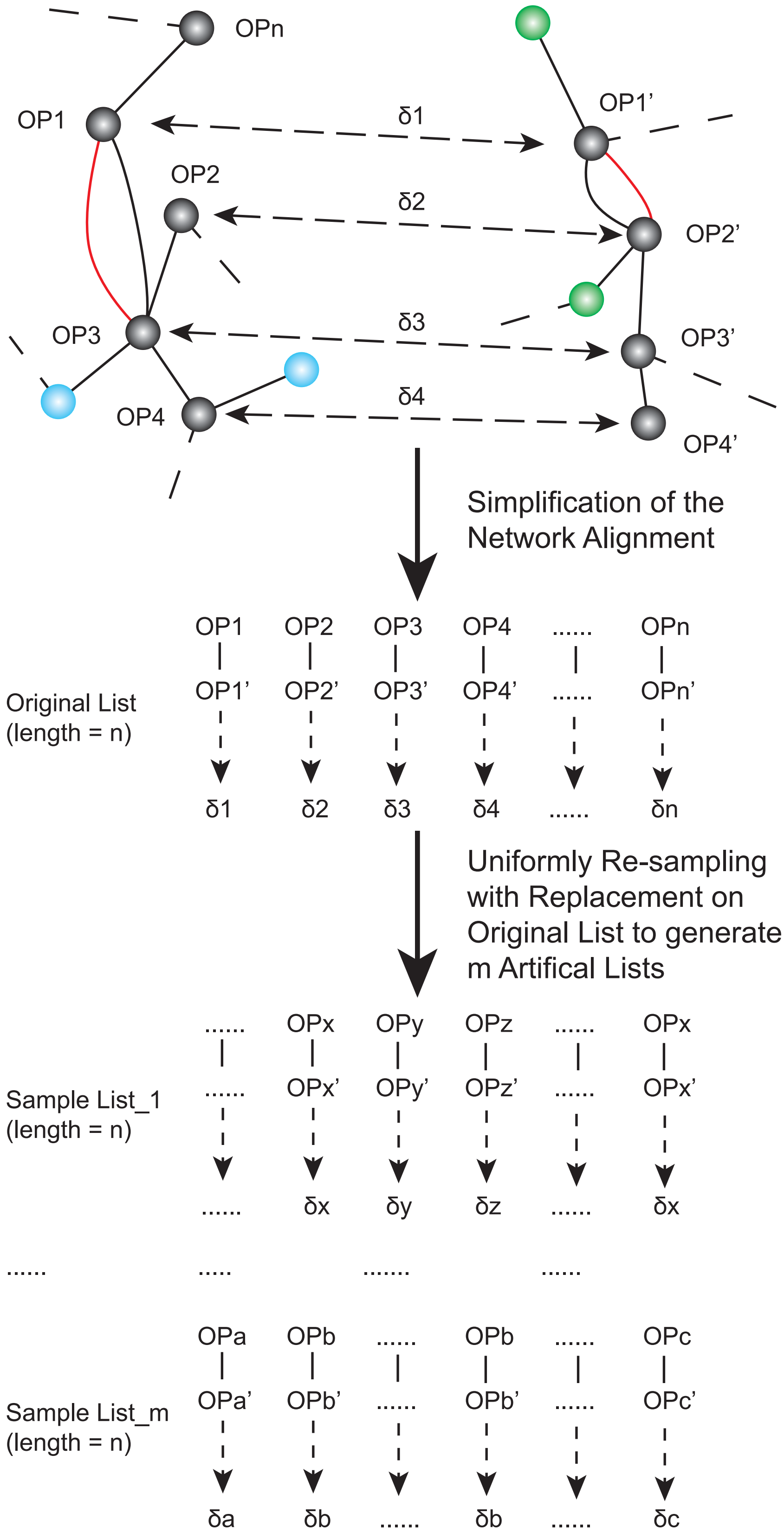
OPn
OP1
δ1
OP1'
OP2
δ2
OP2'
OP3
δ3
OP3'
OP4
δ4
OP4'
Simplification of the Network Alignment
OP1 OP2 OP3 OP4 ...... OPn
OP1' OP2' OP3' OP4' ...... OPn'
Original List (length = n)
δ1 δ2 δ3 δ4 ...... δn
Uniformly Re-sampling with Replacement on Original List to generate m Artifical Lists
...... OPx OPy OPz ...... OPx
...... OPx' OPy' OPz' ...... OPx'
Sample List_1 (length = n)
...... δx δy δz ...... δx
....
OPa OPb ...... OPb ...... OPc
OPa' OPb' ...... OPb' ...... OPc'
Sample List_m (length = n)
δa δb ...... δb ...... δc

Figure S3
