# Supplementary material for: Tree of Life Based on Genome Context Networks
Source: PLoS One. 2008 Oct 9;3(10):e3357. doi: 10.1371/journal.pone.0003357 (PMC2566592; doi:10.1371/journal.pone.0003357)
Supplement: Figure S4 — Rectangular cladogram of phylogenetic tree of 195 representative species. (0.15 MB PDF) [file pone.0003357.s006.pdf]

Figure S4. Rectangular cladogram of phylogenetic tree of 195 representative species.

This figure is the counterpart to the Fig. 2 in the text. For space reason, circle style was used to render the phylogeny of 195 representative species based on genome context networks and the robustness proportion was displayed as the width of branch in Fig. 2 in the text. Therefore, we attached the original phylogenetic tree with exact robustness proportions under the branches.

Figure S4

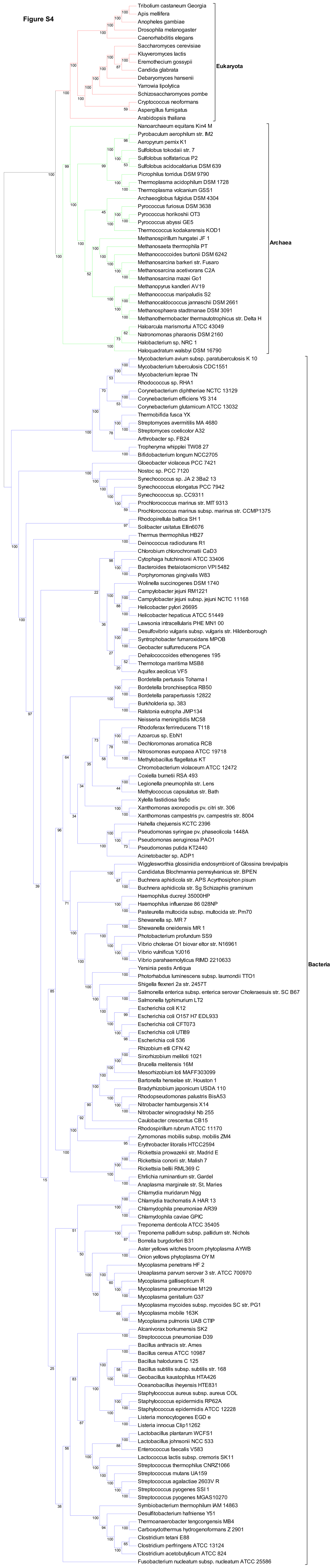
Tribolium castaneum Georgia
Apis mellifera
Anopheles gambiae
Drosophila melanogaster
Caenorhabditis elegans
Saccharomyces cerevisiae
Kluyveromyces lactis
Eremothecium gossypii
Candida glabrata
Debaryomyces hansenii
Yarrowia lipolytica
Schizosaccharomyces pombe
Cryptococcus neoformans
Aspergillus fumigatus
Arabidopsis thaliana
Eukaryota
Nanoarchaeum equitans Kin4 M
Pyrobaculum aerophilum str. IM2
Aeropyrum pernix K1
Sulfolobus tokodaii str. 7
Sulfolobus solfataricus P2
Sulfolobus acidocaldarius DSM 639
Picrophilus torridus DSM 9790
Thermoplasma acidophilum DSM 1728
Thermoplasma volcanium GSS1
Archaeoglobus fulgidus DSM 4304
Pyrococcus furiosus DSM 3638
Pyrococcus horikoshii OT3
Pyrococcus abyssi GE5
Thermococcus kodakarensis KOD1
Methanospirillum hungatei JF 1
Methanosaeta thermophila PT
Methanococcoides burtonii DSM 6242
Methanosarcina barkeri str. Fusaro
Methanosarcina acetivorans C2A
Methanosarcina mazei Go1
Methanopyrus kandleri AV19
Methanococcus maripaludis S2
Methanocaldococcus jannaschii DSM 2661
Methanosphaera stadtmanae DSM 3091
Methanothermobacter thermautotrophicus str. Delta H
Haloarcula marismortui ATCC 43049
Natronomonas pharaonis DSM 2160
Halobacterium sp. NRC 1
Haloquadratum walsbyi DSM 16790
Archaea
Mycobacterium avium subsp. paratuberculosis K 10
Mycobacterium tuberculosis CDC1551
Mycobacterium leprae TN
Rhodococcus sp. RHA1
Corynebacterium diphtheriae NCTC 13129
Corynebacterium efficiens YS 314
Corynebacterium glutamicum ATCC 13032
Thermobifida fusca YX
Streptomyces avermitilis MA 4680
Streptomyces coelicolor A32
Arthrobacter sp. FB24
Tropheryma whipplei TW08 27
Bifidobacterium longum NCC2705
Gloeobacter violaceus PCC 7421
Nostoc sp. PCC 7120
Synechococcus sp. JA 2 3Ba2 13
Synechococcus elongatus PCC 7942
Synechococcus sp. CC9311
Prochlorococcus marinus str. MIT 9313
Prochlorococcus marinus subsp. marinus str. CCMP1375
Rhodopirellula baltica SH 1
Solibacter usitatus Ellin6076
Thermus thermophilus HB27
Deinococcus radiodurans R1
Chlorobium chlorochromatii CaD3
Cytophaga hutchinsonii ATCC 33406
Bacteroides thetaiotaomicron VPI 5482
Porphyromonas gingivalis W83
Wolinella succinogenes DSM 1740
Campylobacter jejuni RM1221
Campylobacter jejuni subsp. jejuni NCTC 11168
Helicobacter pylori 26695
Helicobacter hepaticus ATCC 51449
Lawsonia intracellularis PHE MN1 00
Desulfovibrio vulgaris subsp. vulgaris str. Hildenborough
Syntrophobacter fumaroxidans MPOB
Geobacter sulfurreducens PCA
Dehalococcoides ethenogenes 195
Thermotoga maritima MSB8
Aquifex aeolicus VF5
Bordetella pertussis Tohama I
Bordetella bronchiseptica RB50
Bordetella parapertussis 12822
Burkholderia sp. 383
Ralstonia eutropha JMP134
Neisseria meningitidis MC58
Rhodoferax ferrireducens T118
Azoarcus sp. EbN1
Dechloromonas aromatica RCB
Nitrosomonas europaea ATCC 19718
Methylobacillus flagellatus KT
Chromobacterium violaceum ATCC 12472
Coxiella burnetii RSA 493
Legionella pneumophila str. Lens
Methylococcus capsulatus str. Bath
Xylella fastidiosa 9a5c
Xanthomonas axonopodis pv. citri str. 306
Xanthomonas campestris pv. campestris str. 8004
Hahella chejuensis KCTC 2396
Pseudomonas syringae pv. phaseolicola 1448A
Pseudomonas aeruginosa PAO1
Pseudomonas putida KT2440
Acinetobacter sp. ADP1
Wigglesworthia glossinidia endosymbiont of Glossina brevipalpis
Candidatus Blochmannia pennsylvanicus str. BPEN
Buchnera aphidicola str. APS Acyrthosiphon pisum
Buchnera aphidicola str. Sg Schizaphis graminum
Haemophilus ducreyi 35000HP
Haemophilus influenzae 86 028NP
Pasteurella multocida subsp. multocida str. Pm70
Shewanella sp. MR 7
Shewanella oneidensis MR 1
Photobacterium profundum SS9
Vibrio cholerae O1 biovar eltor str. N16961
Vibrio vulnificus YJ016
Vibrio parahaemolyticus RIMD 2210633
Yersinia pestis Antiqua
Photorhabdus luminescens subsp. laumondii TTO1
Shigella flexneri 2a str. 2457T
Salmonella enterica subsp. enterica serovar Choleraesuis str. SC B67
Salmonella typhimurium LT2
Escherichia coli K12
Escherichia coli O157 H7 EDL933
Escherichia coli CFT073
Escherichia coli UTI89
Escherichia coli 536
Rhizobium etli CFN 42
Sinorhizobium meliloti 1021
Brucella melitensis 16M
Mesorhizobium loti MAFF303099
Bartonella henselae str. Houston 1
Bradyrhizobium japonicum USDA 110
Rhodopseudomonas palustris BisA53
Nitrobacter hamburgensis X14
Nitrobacter winogradskyi Nb 255
Caulobacter crescentus CB15
Rhodospirillum rubrum ATCC 11170
Zymomonas mobilis subsp. mobilis ZM4
Erythrobacter litoralis HTCC2594
Rickettsia prowazekii str. Madrid E
Rickettsia conorii str. Malish 7
Rickettsia bellii RML369 C
Ehrlichia ruminantium str. Gardel
Anaplasma marginale str. St. Maries
Chlamydia muridarum Nigg
Chlamydia trachomatis A HAR 13
Chlamydophila pneumoniae AR39
Chlamydophila caviae GPIC
Treponema denticola ATCC 35405
Treponema pallidum subsp. pallidum str. Nichols
Borrelia burgdorferi B31
Aster yellows witches broom phytoplasma AYWB
Onion yellows phytoplasma OY M
Mycoplasma penetrans HF 2
Ureaplasma parvum serovar 3 str. ATCC 700970
Mycoplasma gallisepticum R
Mycoplasma pneumoniae M129
Mycoplasma genitalium G37
Mycoplasma mycoides subsp. mycoides SC str. PG1
Mycoplasma mobile 163K
Mycoplasma pulmonis UAB CTIP
Alcanivorax borkumensis SK2
Streptococcus pneumoniae D39
Bacillus anthracis str. Ames
Bacillus cereus ATCC 10987
Bacillus halodurans C 125
Bacillus subtilis subsp. subtilis str. 168
Geobacillus kaustophilus HTA426
Oceanobacillus iheyensis HTE831
Staphylococcus aureus subsp. aureus COL
Staphylococcus epidermidis RP62A
Staphylococcus epidermidis ATCC 12228
Listeria monocytogenes EGD e
Listeria innocua Clip11262
Lactobacillus plantarum WCFS1
Lactobacillus johnsonii NCC 533
Enterococcus faecalis V583
Lactococcus lactis subsp. cremoris SK11
Streptococcus thermophilus CNRZ1066
Streptococcus mutans UA159
Streptococcus agalactiae 2603V R
Streptococcus pyogenes SSI 1
Streptococcus pyogenes MGAS10270
Symbiobacterium thermophilum IAM 14863
Desulfitobacterium hafniense Y51
Thermoanaerobacter tengcongensis MB4
Carboxydothermus hydrogenoformans Z 2901
Clostridium tetani E88
Clostridium perfringens ATCC 13124
Clostridium acetobutylicum ATCC 824
Fusobacterium nucleatum subsp. nucleatum ATCC 25586
Bacteria
