# Supplementary material for: Tree of Life Based on Genome Context Networks
Source: PLoS One. 2008 Oct 9;3(10):e3357. doi: 10.1371/journal.pone.0003357 (PMC2566592; doi:10.1371/journal.pone.0003357)
Supplement: Figure S5 — Distribution of genome context work sizes. (0.09 MB PDF) [file pone.0003357.s007.pdf]

Figure S5. Distribution of genome context work sizes.

The exact numbers can be obtained from Supplemental Table 1 and Supplemental Table 2. Red arrow denotes the quantile of 0.95 in this distribution and the value of size is 6917.1. Obviously, network sizes of speices in Deuterostomia (13568.75 ± 4486.674) are significantly higher than normal networks (1786.24 ± 1318.14) ($p < 0.05$), while Actinobacteria (2036.231 ± 1210.759) are as the normal ones ($p > 0.1$).

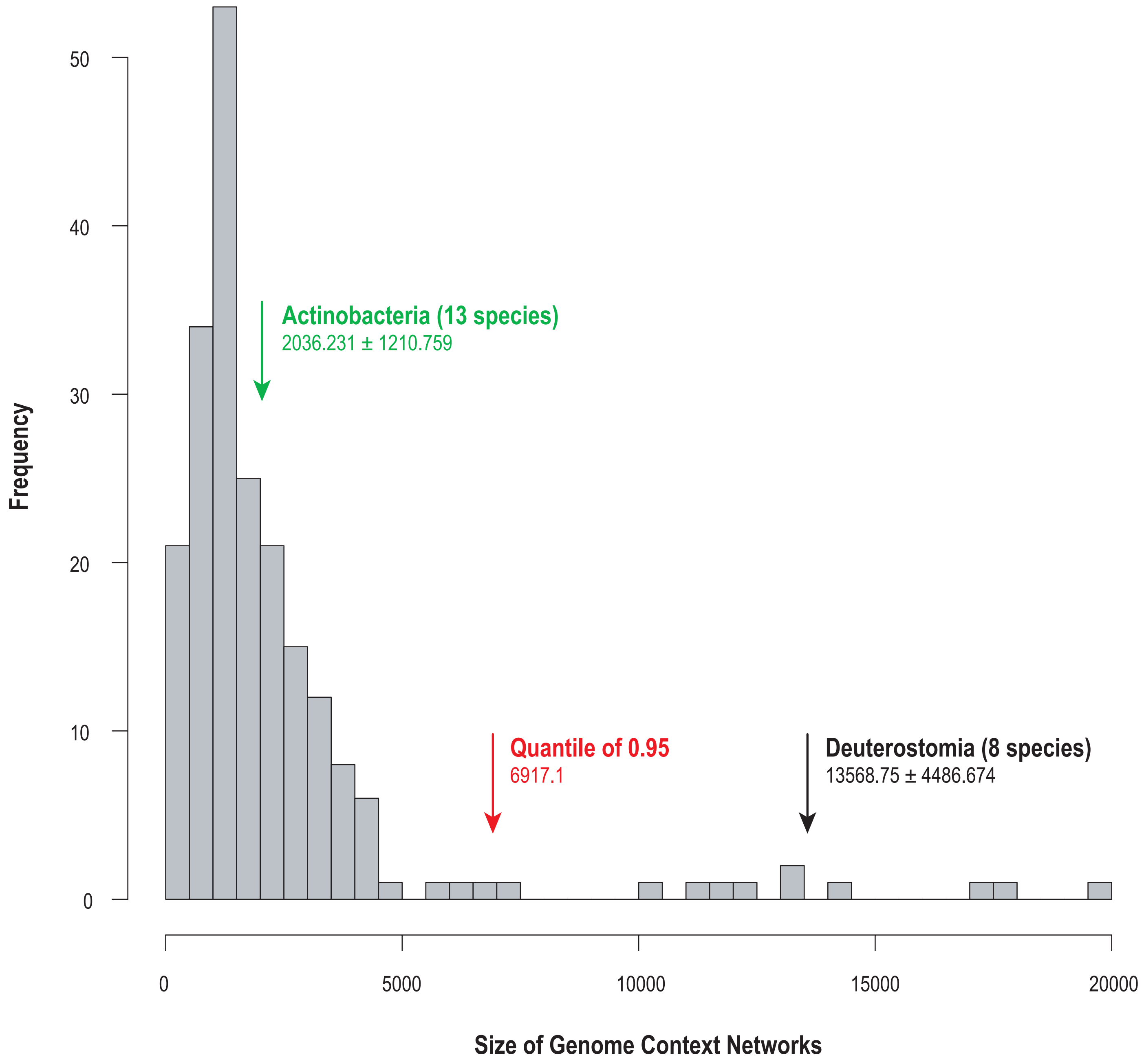

Frequency
0
10
20
30
40
50
Actinobacteria (13 species)
2036.231 ± 1210.759
Quantile of 0.95
6917.1
Deuterostomia (8 species)
13568.75 ± 4486.674
0
5000
10000
15000
20000
Size of Genome Context Networks

Figure S5
