# Supplementary material for: Tree of Life Based on Genome Context Networks
Source: PLoS One. 2008 Oct 9;3(10):e3357. doi: 10.1371/journal.pone.0003357 (PMC2566592; doi:10.1371/journal.pone.0003357)
Supplement: Figure S6 — Phylogenetic tree based on genome context network after addition of Deuterostomia. (0.15 MB PDF) [file pone.0003357.s008.pdf]

Figure S6. Phylogenetic tree based on genome context network after addition of Deuterostomia.

Deuterostomia are highlighted by gray background. Within this clade, the branch order is consistent with current phylogenetic knowledge on the Deuterostomia. However, it was placed as a separate deep branch, which may be an artifact of big network size. Actinobacteria are also highlighted by red background, as they are interested in our work. As expected, main topological features mentioned in the text and Supplemental Table 4 are supported in this phylogenetic tree.

Figure S6

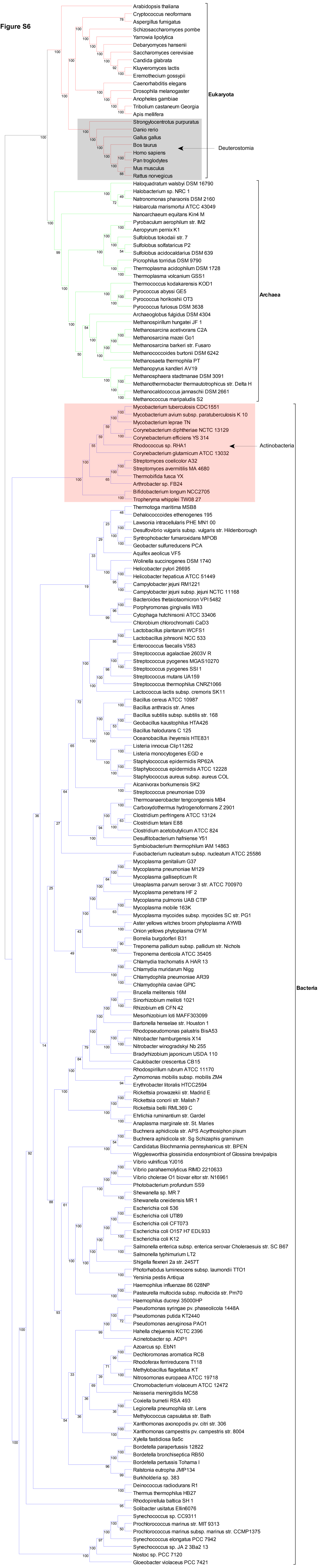

Arabidopsis thaliana
Cryptococcus neoformans
Aspergillus fumigatus
Schizosaccharomyces pombe
Yarrowia lipolytica
Debaryomyces hansenii
Saccharomyces cerevisiae
Candida glabrata
Kluyveromyces lactis
Eremothecium gossypii
Caenorhabditis elegans
Drosophila melanogaster
Anopheles gambiae
Tribolium castaneum Georgia
Apis mellifera
Strongylocentrotus purpuratus
Danio rerio
Gallus gallus
Bos taurus
Homo sapiens
Pan troglodytes
Mus musculus
Rattus norvegicus
Eukaryota
Deuterostomia
Haloquadratum walsbyi DSM 16790
Halobacterium sp. NRC 1
Natronomonas pharaonis DSM 2160
Haloarcula marismortui ATCC 43049
Nanoarchaeum equitans Kin4 M
Pyrobaculum aerophilum str. IM2
Aeropyrum pernix K1
Sulfolobus tokodaii str. 7
Sulfolobus solfataricus P2
Sulfolobus acidocaldarius DSM 639
Picrophilus torridus DSM 9790
Thermoplasma acidophilum DSM 1728
Thermoplasma volcanium GSS1
Thermococcus kodakarensis KOD1
Pyrococcus abyssi GE5
Pyrococcus horikoshii OT3
Pyrococcus furiosus DSM 3638
Archaeoglobus fulgidus DSM 4304
Methanospirillum hungatei JF 1
Methanosarcina acetivorans C2A
Methanosarcina mazei Go1
Methanosarcina barkeri str. Fusaro
Methanococcoides burtonii DSM 6242
Methanosaeta thermophila PT
Methanopyrus kandleri AV19
Methanosphaera stadtmanae DSM 3091
Methanothermobacter thermautotrophicus str. Delta H
Methanocaldococcus jannaschii DSM 2661
Methanococcus maripaludis S2
Archaea
Mycobacterium tuberculosis CDC1551
Mycobacterium avium subsp. paratuberculosis K 10
Mycobacterium leprae TN
Corynebacterium diphtheriae NCTC 13129
Corynebacterium efficiens YS 314
Rhodococcus sp. RHA1
Corynebacterium glutamicum ATCC 13032
Streptomyces coelicolor A32
Streptomyces avermitilis MA 4680
Thermobifida fusca YX
Arthrobacter sp. FB24
Bifidobacterium longum NCC2705
Tropheryma whipplei TW08 27
Actinobacteria
Thermotoga maritima MSB8
Dehalococcoides ethenogenes 195
Lawsonia intracellularis PHE MN1 00
Desulfovibrio vulgaris subsp. vulgaris str. Hildenborough
Syntrophobacter fumaroxidans MPOB
Geobacter sulfurreducens PCA
Aquifex aeolicus VF5
Wolinella succinogenes DSM 1740
Helicobacter pylori 26695
Helicobacter hepaticus ATCC 51449
Campylobacter jejuni RM1221
Campylobacter jejuni subsp. jejuni NCTC 11168
Bacteroides thetaiotaomicron VPI 5482
Porphyromonas gingivalis W83
Cytophaga hutchinsonii ATCC 33406
Chlorobium chlorochromatii CaD3
Lactobacillus plantarum WCFS1
Lactobacillus johnsonii NCC 533
Enterococcus faecalis V583
Streptococcus agalactiae 2603V R
Streptococcus pyogenes MGAS10270
Streptococcus pyogenes SSI 1
Streptococcus mutans UA159
Streptococcus thermophilus CNRZ1066
Lactococcus lactis subsp. cremoris SK11
Bacillus cereus ATCC 10987
Bacillus anthracis str. Ames
Bacillus subtilis subsp. subtilis str. 168
Geobacillus kaustophilus HTA426
Bacillus halodurans C 125
Oceanobacillus iheyensis HTE831
Listeria innocua Clip11262
Listeria monocytogenes EGD e
Staphylococcus epidermidis RP62A
Staphylococcus epidermidis ATCC 12228
Staphylococcus aureus subsp. aureus COL
Alcanivorax borkumensis SK2
Streptococcus pneumoniae D39
Thermoanaerobacter tengcongensis MB4
Carboxydothermus hydrogenoformans Z 2901
Clostridium perfringens ATCC 13124
Clostridium tetani E88
Clostridium acetobutylicum ATCC 824
Desulfitobacterium hafniense Y51
Symbiobacterium thermophilum IAM 14863
Fusobacterium nucleatum subsp. nucleatum ATCC 25586
Mycoplasma genitalium G37
Mycoplasma pneumoniae M129
Mycoplasma gallisepticum R
Ureaplasma parvum serovar 3 str. ATCC 700970
Mycoplasma penetrans HF 2
Mycoplasma pulmonis UAB CTIP
Mycoplasma mobile 163K
Mycoplasma mycoides subsp. mycoides SC str. PG1
Aster yellows witches broom phytoplasma AYWB
Onion yellows phytoplasma OY M
Borrelia burgdorferi B31
Treponema pallidum subsp. pallidum str. Nichols
Treponema denticola ATCC 35405
Chlamydia trachomatis A HAR 13
Chlamydia muridarum Nigg
Chlamydophila pneumoniae AR39
Chlamydophila caviae GPIC
Brucella melitensis 16M
Sinorhizobium meliloti 1021
Rhizobium etli CFN 42
Mesorhizobium loti MAFF303099
Bartonella henselae str. Houston 1
Rhodopseudomonas palustris BisA53
Nitrobacter hamburgensis X14
Nitrobacter winogradskyi Nb 255
Bradyrhizobium japonicum USDA 110
Caulobacter crescentus CB15
Rhodospirillum rubrum ATCC 11170
Zymomonas mobilis subsp. mobilis ZM4
Erythrobacter litoralis HTCC2594
Rickettsia prowazekii str. Madrid E
Rickettsia conorii str. Malish 7
Rickettsia bellii RML369 C
Ehrlichia ruminantium str. Gardel
Anaplasma marginale str. St. Maries
Buchnera aphidicola str. APS Acyrthosiphon pisum
Buchnera aphidicola str. Sg Schizaphis graminum
Candidatus Blochmannia pennsylvanicus str. BPEN
Wigglesworthia glossinidia endosymbiont of Glossina brevipalpis
Vibrio vulnificus YJ016
Vibrio parahaemolyticus RIMD 2210633
Vibrio cholerae O1 biovar eltor str. N16961
Photobacterium profundum SS9
Shewanella sp. MR 7
Shewanella oneidensis MR 1
Escherichia coli 536
Escherichia coli UTI89
Escherichia coli CFT073
Escherichia coli O157 H7 EDL933
Escherichia coli K12
Salmonella enterica subsp. enterica serovar Choleraesuis str. SC B67
Salmonella typhimurium LT2
Shigella flexneri 2a str. 2457T
Photorhabdus luminescens subsp. laumondii TTO1
Yersinia pestis Antiqua
Haemophilus influenzae 86 028NP
Pasteurella multocida subsp. multocida str. Pm70
Haemophilus ducreyi 35000HP
Pseudomonas syringae pv. phaseolicola 1448A
Pseudomonas putida KT2440
Pseudomonas aeruginosa PAO1
Hahella chejuensis KCTC 2396
Acinetobacter sp. ADP1
Azoarcus sp. EbN1
Dechloromonas aromatica RCB
Rhodoferax ferrireducens T118
Methylobacillus flagellatus KT
Nitrosomonas europaea ATCC 19718
Chromobacterium violaceum ATCC 12472
Neisseria meningitidis MC58
Coxiella burnetii RSA 493
Legionella pneumophila str. Lens
Methylococcus capsulatus str. Bath
Xanthomonas axonopodis pv. citri str. 306
Xanthomonas campestris pv. campestris str. 8004
Xylella fastidiosa 9a5c
Bordetella parapertussis 12822
Bordetella bronchiseptica RB50
Bordetella pertussis Tohama I
Ralstonia eutropha JMP134
Burkholderia sp. 383
Deinococcus radiodurans R1
Thermus thermophilus HB27
Rhodopirellula baltica SH 1
Solibacter usitatus Ellin6076
Synechococcus sp. CC9311
Prochlorococcus marinus str. MIT 9313
Prochlorococcus marinus subsp. marinus str. CCMP1375
Synechococcus elongatus PCC 7942
Synechococcus sp. JA 2 3Ba2 13
Nostoc sp. PCC 7120
Gloeobacter violaceus PCC 7421
Bacteria
