# Supplementary material for: Tree of Life Based on Genome Context Networks
Source: PLoS One. 2008 Oct 9;3(10):e3357. doi: 10.1371/journal.pone.0003357 (PMC2566592; doi:10.1371/journal.pone.0003357)
Supplement: Table S1 — Species used in this work. (0.27 MB DOC) [file pone.0003357.s009.doc]

**Table S1.** Species used in this work.

A total of 195 species were analysed, with 29 species of Archaea, 151 of Bacteria and 15 of Eukaryota. For each species, the superkingdom, complete species name, taxonomy ID according to NCBI (<http://www.ncbi.nlm.nih.gov/>) and the size of the genome context network (the number of the nodes in the genome context network) in this study are listed. Note that gene number in the genome is different from the genome context network size, as the genes without any relationships to other genes in the genome context network (*p* < 0.05 for each edge in the networks) will be excluded in the computation of the size of the genome context network.

| **Domain** | **Species Name** | **Taxonomy ID** | **Network Size** |
| --- | --- | --- | --- |
| Archaea | Nanoarchaeum equitans Kin4-M | 228908 | 169 |
| Archaea | Archaeoglobus fulgidus DSM 4304 | 224325 | 1260 |
| Archaea | Haloarcula marismortui ATCC 43049 | 272569 | 1240 |
| Archaea | Halobacterium sp. NRC-1 | 64091 | 768 |
| Archaea | Haloquadratum walsbyi DSM 16790 | 362976 | 971 |
| Archaea | Methanocaldococcus jannaschii DSM 2661 | 243232 | 946 |
| Archaea | Methanococcoides burtonii DSM 6242 | 259564 | 1432 |
| Archaea | Methanopyrus kandleri AV19 | 190192 | 676 |
| Archaea | Methanosarcina acetivorans C2A | 188937 | 2521 |
| Archaea | Methanosarcina barkeri str. Fusaro | 269797 | 2108 |
| Archaea | Methanosarcina mazei Go1 | 192952 | 2007 |
| Archaea | Methanosphaera stadtmanae DSM 3091 | 339860 | 857 |
| Archaea | Aeropyrum pernix K1 | 272557 | 557 |
| Archaea | Methanothermobacter thermautotrophicus str. Delta H | 187420 | 978 |
| Archaea | Methanococcus maripaludis S2 | 267377 | 1068 |
| Archaea | Natronomonas pharaonis DSM 2160 | 348780 | 1063 |
| Archaea | Picrophilus torridus DSM 9790 | 263820 | 788 |
| Archaea | Pyrobaculum aerophilum str. IM2 | 178306 | 814 |
| Archaea | Pyrococcus abyssi GE5 | 272844 | 1151 |
| Archaea | Pyrococcus furiosus DSM 3638 | 186497 | 1181 |
| Archaea | Pyrococcus horikoshii OT3 | 70601 | 1069 |
| Archaea | Sulfolobus acidocaldarius DSM 639 | 330779 | 1052 |
| Archaea | Sulfolobus solfataricus P2 | 273057 | 1444 |
| Archaea | Sulfolobus tokodaii str. 7 | 273063 | 1154 |
| Archaea | Thermococcus kodakarensis KOD1 | 69014 | 1191 |
| Archaea | Thermoplasma acidophilum DSM 1728 | 273075 | 797 |
| Archaea | Thermoplasma volcanium GSS1 | 273116 | 792 |
| Archaea | Methanospirillum hungatei JF-1 | 323259 | 1550 |
| Archaea | Methanosaeta thermophila PT | 349307 | 1077 |
| Bacteria | Bordetella pertussis Tohama I | 257313 | 2556 |
| Bacteria | Azoarcus sp. EbN1 | 76114 | 2228 |
| Bacteria | Aster yellows witches'-broom phytoplasma AYWB | 322098 | 289 |
| Bacteria | Arthrobacter sp. FB24 | 290399 | 2033 |
| Bacteria | Anaplasma marginale str. St. Maries | 234826 | 535 |
| Bacteria | Alcanivorax borkumensis SK2 | 393595 | 1773 |
| Bacteria | Aquifex aeolicus VF5 | 224324 | 762 |
| Bacteria | Bacillus anthracis str. Ames | 198094 | 3117 |
| Bacteria | Bacillus cereus ATCC 10987 | 222523 | 3179 |
| Bacteria | Bacillus halodurans C-125 | 272558 | 2553 |
| Bacteria | Bacillus subtilis subsp. subtilis str. 168 | 224308 | 2622 |
| Bacteria | Bacteroides thetaiotaomicron VPI-5482 | 226186 | 2031 |
| Bacteria | Bifidobacterium longum NCC2705 | 206672 | 795 |
| Bacteria | Burkholderia sp. 383 | 269483 | 4478 |
| Bacteria | Campylobacter jejuni RM1221 | 195099 | 1003 |
| Bacteria | Acinetobacter sp. ADP1 | 62977 | 2064 |
| Bacteria | Chlamydophila pneumoniae AR39 | 115711 | 465 |
| Bacteria | Chlamydophila caviae GPIC | 227941 | 469 |
| Bacteria | Chlamydia trachomatis A/HAR-13 | 315277 | 422 |
| Bacteria | Chlamydia muridarum Nigg | 243161 | 425 |
| Bacteria | Bordetella bronchiseptica RB50 | 257310 | 3448 |
| Bacteria | Campylobacter jejuni subsp. jejuni NCTC 11168 | 192222 | 1002 |
| Bacteria | Bordetella parapertussis 12822 | 257311 | 2978 |
| Bacteria | Buchnera aphidicola str. Sg (Schizaphis graminum) | 198804 | 485 |
| Bacteria | Buchnera aphidicola str. APS (Acyrthosiphon pisum) | 107806 | 507 |
| Bacteria | Brucella melitensis 16M | 224914 | 2025 |
| Bacteria | Bradyrhizobium japonicum USDA 110 | 224911 | 4327 |
| Bacteria | Borrelia burgdorferi B31 | 224326 | 350 |
| Bacteria | Carboxydothermus hydrogenoformans Z-2901 | 246194 | 1481 |
| Bacteria | Candidatus Blochmannia pennsylvanicus str. BPEN | 291272 | 572 |
| Bacteria | Clostridium perfringens ATCC 13124 | 195103 | 1609 |
| Bacteria | Rhodospirillum rubrum ATCC 11170 | 269796 | 2005 |
| Bacteria | Rickettsia bellii RML369-C | 336407 | 655 |
| Bacteria | Shewanella sp. MR-7 | 60481 | 2564 |
| Bacteria | Streptococcus thermophilus CNRZ1066 | 299768 | 1178 |
| Bacteria | Symbiobacterium thermophilum IAM 14863 | 292459 | 1599 |
| Bacteria | Syntrophobacter fumaroxidans MPOB | 335543 | 1880 |
| Bacteria | Thermobifida fusca YX | 269800 | 1362 |
| Bacteria | Zymomonas mobilis subsp. mobilis ZM4 | 264203 | 1043 |
| Bacteria | Rhodoferax ferrireducens T118 | 338969 | 2631 |
| Bacteria | Rhodococcus sp. RHA1 | 101510 | 3667 |
| Bacteria | Rhizobium etli CFN 42 | 347834 | 2610 |
| Bacteria | Ralstonia eutropha JMP134 | 264198 | 3749 |
| Bacteria | Nitrobacter winogradskyi Nb-255 | 323098 | 1905 |
| Bacteria | Bartonella henselae str. Houston-1 | 283166 | 1028 |
| Bacteria | Geobacillus kaustophilus HTA426 | 235909 | 2222 |
| Bacteria | Caulobacter crescentus CB15 | 190650 | 1836 |
| Bacteria | Chlorobium chlorochromatii CaD3 | 340177 | 984 |
| Bacteria | Cytophaga hutchinsonii ATCC 33406 | 269798 | 1386 |
| Bacteria | Dechloromonas aromatica RCB | 159087 | 2463 |
| Bacteria | Desulfitobacterium hafniense Y51 | 138119 | 2779 |
| Bacteria | Nitrobacter hamburgensis X14 | 323097 | 2114 |
| Bacteria | Erythrobacter litoralis HTCC2594 | 314225 | 1461 |
| Bacteria | Methylococcus capsulatus str. Bath | 243233 | 1652 |
| Bacteria | Hahella chejuensis KCTC 2396 | 349521 | 3094 |
| Bacteria | Lawsonia intracellularis PHE/MN1-00 | 363253 | 680 |
| Bacteria | Legionella pneumophila str. Lens | 297245 | 1487 |
| Bacteria | Clostridium acetobutylicum ATCC 824 | 272562 | 1961 |
| Bacteria | Methylobacillus flagellatus KT | 265072 | 1812 |
| Bacteria | Rhodopirellula baltica SH 1 | 243090 | 1545 |
| Bacteria | Ehrlichia ruminantium str. Gardel | 302409 | 536 |
| Bacteria | S. enterica subsp. enterica serovar Choleraesuis | 321314 | 3500 |
| Bacteria | Streptococcus mutans UA159 | 210007 | 1325 |
| Bacteria | Streptococcus agalactiae 2603V/R | 208435 | 1457 |
| Bacteria | Staphylococcus epidermidis RP62A | 176279 | 1624 |
| Bacteria | Staphylococcus epidermidis ATCC 12228 | 176280 | 1612 |
| Bacteria | Staphylococcus aureus subsp. aureus COL | 93062 | 1706 |
| Bacteria | Solibacter usitatus Ellin6076 | 234267 | 2985 |
| Bacteria | Sinorhizobium meliloti 1021 | 266834 | 2295 |
| Bacteria | Shigella flexneri 2a str. 2457T | 198215 | 3602 |
| Bacteria | Photorhabdus luminescens subsp. laumondii TTO1 | 243265 | 2995 |
| Bacteria | Salmonella typhimurium LT2 | 99287 | 3745 |
| Bacteria | Streptococcus pyogenes SSI-1 | 193567 | 1283 |
| Bacteria | Rickettsia prowazekii str. Madrid E | 272947 | 521 |
| Bacteria | Rickettsia conorii str. Malish 7 | 272944 | 547 |
| Bacteria | Rhodopseudomonas palustris BisA53 | 316055 | 2664 |
| Bacteria | Pseudomonas syringae pv. phaseolicola 1448A | 264730 | 3166 |
| Bacteria | Pseudomonas putida KT2440 | 160488 | 3461 |
| Bacteria | Pseudomonas aeruginosa PAO1 | 208964 | 3563 |
| Bacteria | Prochlorococcus marinus str. CCMP1375 | 167539 | 998 |
| Bacteria | Prochlorococcus marinus str. MIT 9313 | 74547 | 1093 |
| Bacteria | Chromobacterium violaceum ATCC 12472 | 243365 | 2372 |
| Bacteria | Shewanella oneidensis MR-1 | 211586 | 2673 |
| Bacteria | Treponema denticola ATCC 35405 | 243275 | 1001 |
| Bacteria | Xylella fastidiosa 9a5c | 160492 | 1264 |
| Bacteria | Xanthomonas campestris pv. campestris str. 8004 | 314565 | 2243 |
| Bacteria | Xanthomonas axonopodis pv. citri str. 306 | 190486 | 2283 |
| Bacteria | Wolinella succinogenes DSM 1740 | 273121 | 1167 |
| Bacteria | W. glossinidia endosymbiont of Glossina brevipalpis | 36870 | 506 |
| Bacteria | Vibrio vulnificus YJ016 | 196600 | 2972 |
| Bacteria | Vibrio parahaemolyticus RIMD 2210633 | 223926 | 3041 |
| Bacteria | Vibrio cholerae O1 biovar eltor str. N16961 | 243277 | 2492 |
| Bacteria | Ureaplasma parvum serovar 3 str. ATCC 700970 | 273119 | 270 |
| Bacteria | Streptococcus pneumoniae D39 | 373153 | 1326 |
| Bacteria | Treponema pallidum subsp. pallidum str. Nichols | 243276 | 357 |
| Bacteria | Streptococcus pyogenes MGAS10270 | 370552 | 1296 |
| Bacteria | Thermus thermophilus HB27 | 262724 | 873 |
| Bacteria | Thermotoga maritima MSB8 | 243274 | 1033 |
| Bacteria | Thermoanaerobacter tengcongensis MB4 | 273068 | 1591 |
| Bacteria | Synechococcus sp. JA-2-3B'a(2-13) | 321332 | 1375 |
| Bacteria | Synechococcus sp. CC9311 | 64471 | 1180 |
| Bacteria | Synechococcus elongatus PCC 7942 | 1140 | 1339 |
| Bacteria | Streptomyces coelicolor A3(2) | 100226 | 3091 |
| Bacteria | Streptomyces avermitilis MA-4680 | 227882 | 2999 |
| Bacteria | Photobacterium profundum SS9 | 298386 | 3375 |
| Bacteria | Tropheryma whipplei TW08/27 | 218496 | 445 |
| Bacteria | Escherichia coli 536 | 362663 | 3912 |
| Bacteria | Helicobacter hepaticus ATCC 51449 | 235279 | 931 |
| Bacteria | Haemophilus influenzae 86-028NP | 281310 | 1321 |
| Bacteria | Haemophilus ducreyi 35000HP | 233412 | 1138 |
| Bacteria | Gloeobacter violaceus PCC 7421 | 251221 | 1859 |
| Bacteria | Geobacter sulfurreducens PCA | 243231 | 1676 |
| Bacteria | Fusobacterium nucleatum subsp. nucleatum ATCC 25586 | 190304 | 1074 |
| Bacteria | Escherichia coli UTI89 | 364106 | 4023 |
| Bacteria | Escherichia coli O157:H7 EDL933 | 155864 | 4205 |
| Bacteria | Porphyromonas gingivalis W83 | 242619 | 827 |
| Bacteria | Escherichia coli CFT073 | 199310 | 4072 |
| Bacteria | Lactobacillus plantarum WCFS1 | 220668 | 1814 |
| Bacteria | Enterococcus faecalis V583 | 226185 | 1822 |
| Bacteria | Desulfovibrio vulgaris str. Hildenborough | 882 | 1333 |
| Bacteria | Deinococcus radiodurans R1 | 243230 | 1000 |
| Bacteria | Dehalococcoides ethenogenes 195 | 243164 | 875 |
| Bacteria | Coxiella burnetii RSA 493 | 227377 | 978 |
| Bacteria | Corynebacterium glutamicum ATCC 13032 | 196627 | 4507 |
| Bacteria | Corynebacterium efficiens YS-314 | 196164 | 1421 |
| Bacteria | Corynebacterium diphtheriae NCTC 13129 | 257309 | 1230 |
| Bacteria | Clostridium tetani E88 | 212717 | 1394 |
| Bacteria | Escherichia coli K12 | 83333 | 3808 |
| Bacteria | Mycoplasma genitalium G37 | 243273 | 262 |
| Bacteria | Pasteurella multocida subsp. multocida str. Pm70 | 272843 | 1590 |
| Bacteria | Onion yellows phytoplasma OY-M | 262768 | 389 |
| Bacteria | Oceanobacillus iheyensis HTE831 | 221109 | 2351 |
| Bacteria | Nostoc sp. PCC 7120 | 103690 | 2310 |
| Bacteria | Nitrosomonas europaea ATCC 19718 | 228410 | 1602 |
| Bacteria | Neisseria meningitidis MC58 | 122586 | 1205 |
| Bacteria | Mycoplasma pulmonis UAB CTIP | 272635 | 318 |
| Bacteria | Mycoplasma pneumoniae M129 | 272634 | 312 |
| Bacteria | Mycoplasma penetrans HF-2 | 272633 | 461 |
| Bacteria | Helicobacter pylori 26695 | 85962 | 801 |
| Bacteria | Mycoplasma mobile 163K | 267748 | 294 |
| Bacteria | Lactobacillus johnsonii NCC 533 | 257314 | 1099 |
| Bacteria | Mycoplasma gallisepticum R | 233150 | 314 |
| Bacteria | Mycobacterium tuberculosis CDC1551 | 83331 | 1778 |
| Bacteria | Mycobacterium leprae TN | 272631 | 966 |
| Bacteria | Mycobacterium avium subsp. paratuberculosis K-10 | 262316 | 2177 |
| Bacteria | Mesorhizobium loti MAFF303099 | 266835 | 3691 |
| Bacteria | Listeria monocytogenes EGD-e | 169963 | 2028 |
| Bacteria | Listeria innocua Clip11262 | 272626 | 2055 |
| Bacteria | Lactococcus lactis subsp. cremoris SK11 | 272622 | 1474 |
| Bacteria | Yersinia pestis Antiqua | 360102 | 3001 |
| Bacteria | Mycoplasma mycoides subsp. mycoides SC str. PG1 | 272632 | 414 |
| Eukaryota | Anopheles gambiae | 180454 | 4351 |
| Eukaryota | Apis mellifera | 7460 | 1668 |
| Eukaryota | Tribolium castaneum Georgia | 7070 | 3053 |
| Eukaryota | Aspergillus fumigatus | 330879 | 1538 |
| Eukaryota | Arabidopsis thaliana | 3702 | 11271 |
| Eukaryota | Caenorhabditis elegans | 6239 | 6564 |
| Eukaryota | Drosophila melanogaster | 7227 | 5923 |
| Eukaryota | Candida glabrata | 284593 | 1286 |
| Eukaryota | Cryptococcus neoformans | 214684 | 833 |
| Eukaryota | Debaryomyces hansenii | 284592 | 1364 |
| Eukaryota | Eremothecium gossypii | 33169 | 992 |
| Eukaryota | Kluyveromyces lactis | 284590 | 1184 |
| Eukaryota | Saccharomyces cerevisiae | 4932 | 1750 |
| Eukaryota | Schizosaccharomyces pombe | 284812 | 1259 |
| Eukaryota | Yarrowia lipolytica | 284591 | 1311 |
