# Supplementary material for: Tree of Life Based on Genome Context Networks
Source: PLoS One. 2008 Oct 9;3(10):e3357. doi: 10.1371/journal.pone.0003357 (PMC2566592; doi:10.1371/journal.pone.0003357)
Supplement: Table S2 — Species of Deuterostomia used in the big network attraction experiment. (0.04 MB DOC) [file pone.0003357.s010.doc]

**Table S2.** Species of Deuterostomia used in the big network attraction experiment.

The structure of this table is the same as Table S1.

| **Domain** | **Species Name** | **Taxonomy ID** | **Network Size** |
| --- | --- | --- | --- |
| Eukaryota | Bos Taurus | 9913 | 12038 |
| Eukaryota | Gallus gallus | 9031 | 6080^a^ |
| Eukaryota | Pan troglodytes | 9598 | 14131 |
| Eukaryota | Rattus norvegicus | 10116 | 17691 |
| Eukaryota | Strongylocentrotus purpuratus | 7668 | 10139 |
| Eukaryota | Homo sapiens | 9606 | 17194 |
| Eukaryota | Mus musculus | 10090 | 19620 |
| Eukaryota | Danio rerio | 7955 | 11657 |

^a^ The status of Chick genome project was draft assembly when we downloaded this genome Oct., 2006. Comparative analysis reveals that Chick has a compact genome structure containing a similar number of genes as found in mammals but with shorter intergenic DNA sequences and fewer repeats [1]. Therefore, the genome architecture of Chick is greatly different from these of other mammalian Amniotes and the size of its network tended to exceptionally small. However, the cluster of Chick with other mammalian Amniotes (RP = 100%) indicates the existence of a common genome architecture in all Amniotes.

1. Consortium ICGS (2004) Sequence and comparative analysis of the chicken genome provide unique perspectives on vertebrate evolution. Nature 432: 695-716.
