# Supplementary material for: Tree of Life Based on Genome Context Networks
Source: PLoS One. 2008 Oct 9;3(10):e3357. doi: 10.1371/journal.pone.0003357 (PMC2566592; doi:10.1371/journal.pone.0003357)
Supplement: Table S3 — Edge numbers in genome context networks. (0.03 MB DOC) [file pone.0003357.s011.doc]

**Table S3.** Edge numbers in genome context networks.

Edges whose *p* values are less than 0.05 were used to construct the tree of life (Figure 2 in text). We found that the main contribution to construct gene networks is gene neighbors method and relaxing the cutoff of *p* vlaue can increase the contribution of the gene neighbors method (76%, *p* < 0.01; 89%, *p* < 0.05; 95%, *p* < 0.1). However, the main results based on edges with *p* value less than 0.01 or 0.1 are the same as that from less than 0.05.

| **Method used to infer edges** | **Edge *p* value** | | |
| --- | --- | --- | --- |
|  | *p* < 0.01 | *p* < 0.05 | *p* < 0.1 |
| Phylogenetic Profiles | 312815 | 552017 | 576018 |
| Gene Fusions | 218987 | 237006 | 250838 |
| Gene Neighbors | 1680111 | 6295224 | 14764335 |
| SUM | 2211913 | 7084247 | 15591191 |
