# Supplementary material for: Tree of Life Based on Genome Context Networks
Source: PLoS One. 2008 Oct 9;3(10):e3357. doi: 10.1371/journal.pone.0003357 (PMC2566592; doi:10.1371/journal.pone.0003357)
Supplement: Table S4 — Evidence to support the monophyly of each sub-division. (0.09 MB DOC) [file pone.0003357.s012.doc]

**Table S4.** Evidence to support the monophyly of each sub-division.

This table is the counterpart to the Table 1 in the text. It presents more detailed descriptions for controversial sub-divisions.

| **Domain** | **Sub-division** | **RP**^a^ **(%)** |
| --- | --- | --- |
| Eukaryota | Metazoa^b^ | 100 |
|  | Fungi | 100 |
|  | Plantae | -^c^ |
| Archaea | Nanoarchaeota | -^c^ |
|  | Crenarchaeota | 99 |
|  | Euryarchaeota (excluding Halobacteriaceae)^d^ | 100 |
|  | Halobacteriaceae (a family of Euryarchaeota)^d^ | 100 |
| Bacteria | Actinobacteria | 100 |
|  | Cyanobacteria^e^ | 100 |
|  | Planctomycetes | -^c^ |
|  | Firbrobacteres | -^c^ |
|  | Peinococeus | 100 |
|  | Bacteroidetes | 100 |
|  | ε-proteobacteria | 100 |
|  | δ-proteobacteria | 100 |
|  | Chloroflexi | -^c^ |
|  | Thermotogae^f^ | -^c^ |
|  | Aquificae^f^ | -^c^ |
|  | β-proteobacteria^g^ | 64 |
|  | γ-proteobacteria^g^ | 96 |
|  | α-proteobacteria | 100 |
|  | Chlamydiae | 100 |
|  | Spirochaetes | 100 |
|  | Fusobacteria | -^c^ |
|  | Mollicutes (a family of Firmicutes)^h^ | 100 |
|  | Firmicutes (excluding Mollicutes)^h^ | 56 |

^a^ RP stands for “Robustness Proportion”.

^b^ Deuterostomia were excluded here due to their big network sizes (Supplementary Fig. 5 online). In the big network attraction experiment, Deuterostomia were clustered as separated group (RP = 100%) and placed at the deep branch before Fungi and after Plantae (RP = 100%) (Supplementary Fig. 6 online).

^c^ Only one species of the phylum was used in our study. Hence, we can’t assess the monophyly of this phylum here.

^d^ The family of Halobacteriaceae in Euryarchaeota are suggested as the deepest branch in Archaea in our tree (RP = 100%). Thereby our result disrupts the monophyly of Euryarchaeota. However, excluding Halobacteriaceae, the remain topological features are consistent with the framework of early Archaeal evolution suggested in previous work [1]. These results heat up the controversy of history in Archaeal domain, such as the position of Nanoarchaeota [2,3].

^e^ The monophyletic photosynthetic Bacteria of Cyanobacteria are placed at the deep branch of the Bacteria [4] after Actinobacteria (RP = 100%), indicating an early occurrence of oxygenic photosynthesis which is an important result for both biology and geochemistry [5]. Given credible fossil data for calibration, it is theoretically possible to date the age for Cyanobacteria based on our tree [6].

^f^ Surprisingly, Thermotogae, Chloroflexi and Aquificae are grouped together too, albeit with weaker statistical support (RP < 40%). Considering no point mutation information used in our strategy, the grouping of Thermotoga and Aquifex can’t be explained as the result of the compositional bias of primary sequences [3,7] and thus puts forward the question of correlation of the core relationships of gene networks and the life-styles [8].

^g^ Cluster of families of Xanthomonadaceae, Legionellales and Pseudomonadales in γ-proteobacteria and families of Neisseriaceae and Rhodocyclaceae in β-proteobacteria are supported by RP = 34% and then grouped with family of Burkholderiales in β-proteobacteria (RP = 64%), indicating that families of Xanthomonadaceae, Legionellales and Pseudomonadales may be divergent families in β-proteobacteria rather than γ-proteobacteria. In addition, γ-proteobacteria and β-proteobacteria are well grouped together (RP = 96%), in agreement with previous phylogenomical studies [3,9,10].

^h^ Spirochaetes are placed as a sister group of the family of Mollicutes in Firmicutes (RP = 50%). Then, the clade is clustered with Chlamydiae (RP = 51%). Note that, the close relationship between Spirochaetes and Chlamydiae (RP = 51%) is also suggested by [9-11]. Howbeit, the group of Spirochaetes, Chlamydiae and Mollicutes may be an artifact due to small network size (in the 10% smallest genome networks) and parasitic nature of these species.

1. Waters E, Hohn MJ, Ahel I, Graham DE, Adams MD, et al. (2003) The genome of Nanoarchaeum equitans: insights into early archaeal evolution and derived parasitism. Proc Natl Acad Sci U S A 100: 12984-12988.

2. Brochier C, Gribaldo S, Zivanovic Y, Confalonieri F, Forterre P (2005) Nanoarchaea: representatives of a novel archaeal phylum or a fast-evolving euryarchaeal lineage related to Thermococcales? Genome Biol 6: R42.

3. Ciccarelli FD, Doerks T, von Mering C, Creevey CJ, Snel B, et al. (2006) Toward automatic reconstruction of a highly resolved tree of life. Science 311: 1283-1287.

4. Fukami-Kobayashi K, Minezaki Y, Tateno Y, Nishikawa K (2007) A Tree of Life Based on Protein Domain Organizations. Mol Biol Evol.

5. Allen JF, Martin W (2007) Evolutionary biology: out of thin air. Nature 445: 610-612.

6. Welch JJ, Bromham L (2005) Molecular dating when rates vary. Trends Ecol Evol 20: 320-327.

7. Kreil DP, Ouzounis CA (2001) Identification of thermophilic species by the amino acid compositions deduced from their genomes. Nucleic Acids Res 29: 1608-1615.

8. Raymond J, Segre D (2006) The effect of oxygen on biochemical networks and the evolution of complex life. Science 311: 1764-1767.

9. Brown JR, Douady CJ, Italia MJ, Marshall WE, Stanhope MJ (2001) Universal trees based on large combined protein sequence data sets. Nat Genet 28: 281-285.

10. Olsen GJ, Woese CR, Overbeek R (1994) The winds of (evolutionary) change: breathing new life into microbiology. J Bacteriol 176: 1-6.

11. Daubin V, Gouy M, Perriere G (2002) A phylogenomic approach to bacterial phylogeny: evidence of a core of genes sharing a common history. Genome Res 12: 1080-1090.
