# Supplementary material for: Tree of Life Based on Genome Context Networks
Source: PLoS One. 2008 Oct 9;3(10):e3357. doi: 10.1371/journal.pone.0003357 (PMC2566592; doi:10.1371/journal.pone.0003357)
Supplement: Table S5 — Comparison of Gene Content/Networks Based methods in methodology (0.27 MB PDF) [file pone.0003357.s013.pdf]

Table S5

Comparison of Gene Content/Networks Based methods in methodology

<table>
<tr><th>Method
Factor</th><th>Gene Content Based Method</th><th>Gene Networks Based Method</th></tr>
<tr><td>Key Steps</td>
<td>1) Orthologous gene detection.
Most of the methods now are based on bio-sequences, e.g., best-hit method [1], COG method [3].

2) Binary pattern conversion (genome alignment, similar to gene alignment).
<code>AB-DEFGHI</code>
<code>|| || |||</code>
<code>ABCDE-GHI</code>
The order of the gene in this present/basent profile is arbitary [1, 3-5], that is, the genes (or orthologous genes) are independent of others.</td>
<td>1) Gene network construction.
There are many methods to construct a gene nework for a speical organim. But it is difficult to construct the comparabale networks for several species. Therefore, KEGG networks [6] is preferred these days [7], even though they also are manually selected networks.

2) Gene network alignment.
Two existed gene networks can be aligned as they evolved from a last common ancestor which possessed a ommon ancestral network. The purpose of gene network alignment is to find shadow of the last common ancestral networks (LCAN) from two networks. If two vertexes from tow networks are derived from the same node in the LCAN, high score will obtained. In practice, bipartite graph can be used to depict the alignment of two networks. Several algorithms has been developped for the alingment, e.g., Heymans's method [8], Græmlin [9] and so on.
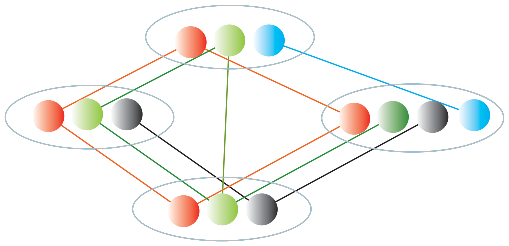
If we did not know too much about the evolution of the vertexes (e.g., internet network), the de nove method is to generate a matrix to depict the similarities of vertexes from these two networks, and then to search this matrix, namely ipartite graph matching [8]. A pivotal step here is to define the vertex similarities in network level.
Two most common definitions are used here: structural equivalence (e.g., Jaccard index) and regular equivalence [2]. The calculation for regular equivalence consumes much computer time as it is using a recursion method [2, 8]. Therefore, structural equivalence is also pupular these days [8, 10].
If we have had some knowledges of the evolution of the vertexes (e.g., gene networks), we can simplify the process of the network alignment. For example, Græmlin [9] and several phylogeny analyses based on KEGG pathways [8, 10]. Based on vertexes' information, we can give a pre-aligment of two networks. An example is that the networks were aligned by orthologs directly [8, 10].
Even though the netowrk alignment can be convert to bipartite graph, the genes in the alignment depend on others, that is, there are relionships with other genes in the network. This character will contribute to the calculation of the similarity of two networks.</td></tr>
</table>

Table S5 (continue)

Factor \ Method	Gene Content Based Method	Gene Networks Based Method
**Key Steps**	**3) Distance measure base on binary pattern of present/absent profiles and build a distance matrix.**<br>Two directions here,<br>one is **the *ad hoc* distance measure**, such as d(G1, G2) = 1 - |G1 ∩ G2| / min(|G1|, |G2|) in Snel's paper [1],<br>the orther is the **model based distance measure**, such as maximum likelihood distance in Huson's paper [11].<br>Note that if use other methods to construct the phylogeny, (e.g.,), this distance should not be calculated but view the orthologous gene group as genetic traits [3].<br>**4) Phylogeny construction based the distance matrix.**<br>Neighbor joining method [12] is the most popular method for phylogeny construction these days.	**3) Distance measurement between networks.**<br>A common method is to sum the structural similarity of mateched vertexes (or genes) after bipartite graph matching [7, 8, 10]. This value should be normalized due to the broad range of network sizes .<br>**4) Phylogeny construction based the distance matrix.**<br>Neighbor joining method [12] is the most popular method for phylogeny construction these days.
**History of the Application Cases of these methods***	**1999** Fitz-Gibbon et.al (11 species, based onbased on the observed presence and absence of families) [13]; Snel et.al. (13 species, based on shared genes) [1]; Tekaia et. al. (20 species, based on shared genes but updated) [14]<br>2000 Montague et.al. (13 herpesviruses, based on COG-like gene family) [3]; Lin et. al. (based on particular molecular features, ortholog and folds) [15];<br>2002 House et. al. (27 species, same as Fitz-Gibbon's method [13]) [16]; Wolf et. al. (57 species, with COG data; give a review of gene content method)[4]; Korbel et. al. (a web server, the parameters can be changed) [17]<br>2004 Huson et. al. (model based distance) [11]; Lake et. al. (conditioned reconstruction) [18]; Rivera et. al. (ring of life, based on Lake 's method[18]) [19]<br>2005 Delsuc et. al. (a review on the phylogenomics) [5]<br>........	1999 Forst et. al (compare the repertoire of a single pathway, include the graph topology) [20]<br>2001 Podani et. al. (biochemical reaction pathways, aligned by substrates and enzymes directly and then mathematical transform) [24]<br>2002 Liao et. al (compre the pathway repertoire, similar to gene content method) (conference paper);<br>**2003** Heymans et. al (defined the similarity of two graph and applied it on metabolic pathways, based on regular equivalence) [8]<br>2004 Hong et. al (reaction content of entire pathways, similar to gene content method) [21]; Ma et. al (based on structural equivalence, metabolic pathways) [10];<br>2006 Zhang et. al (use metabolic pathways, based on structural equivalence) [7]; Forst et. al (use metabolic pathways, based on topology) [22];<br>........

* Maybe we overlook some cases.

Table S5 (continue)

Method / Factor	Gene Content Based Method	Gene Networks Based Method
**Components in the distance measure**	1) Number of shared genes (this is the main contribution for the final distance)<br>2) Numbers of genes in first and second genomes (this component will be weakened due to different normalization method)	1) Network context of the matched vertexes (if structural equivalence, the number of shared gene relationships; this is the main contribution for the final distance)<br>2) Numbers of matched vertexes, size of the both genomes (these components will be weakened due to normalization)
**Biological Sense**	Measure of the conservation of the shared genes	Measure of the conservation of the gene relationships (e.g., genome context) (note that, two genes can have many different relationships)

References (1-24)

1. Snel, B., Bork, P. and Huynen, M.A. (1999) Genome phylogeny based on gene content. *Nat Genet*, **21**, 108-110.
2. Leicht, E.A., Holme, P. and Newman, M.E. (2006) Vertex similarity in networks. *Phys Rev E Stat Nonlin Soft Matter Phys*, **73**, 026120.
3. Montague, M.G. and Hutchison, C.A., 3rd. (2000) Gene content phylogeny of herpesviruses. *Proc Natl Acad Sci U S A*, **97**, 5334-5339.
4. Wolf, Y.I., Rogozin, I.B., Grishin, N.V. and Koonin, E.V. (2002) Genome trees and the tree of life. *Trends Genet*, **18**, 472-479.
5. Delsuc, F., Brinkmann, H. and Philippe, H. (2005) Phylogenomics and the reconstruction of the tree of life. *Nat Rev Genet*, **6**, 361-375.
6. Kanehisa, M. and Goto, S. (2000) KEGG: kyoto encyclopedia of genes and genomes. *Nucleic Acids Res*, **28**, 27-30.
7. Zhang, Y., Li, S., Skogerbo, G., Zhang, Z., Zhu, X., Sun, S., Lu, H., Shi, B. and Chen, R. (2006) Phylophenetic properties of metabolic pathway topologies as revealed by global analysis. *BMC Bioinformatics*, **7**, 252.
8. Heymans, M. and Singh, A.K. (2003) Deriving phylogenetic trees from the similarity analysis of metabolic pathways. *Bioinformatics*, **19 Suppl 1**, i138-146.
9. Flannick, J., Novak, A., Srinivasan, B.S., McAdams, H.H. and Batzoglou, S. (2006) Graemlin: general and robust alignment of multiple large interaction networks. *Genome Res*, **16**, 1169-1181.
10. Ma, H.W. and Zeng, A.P. (2004) Phylogenetic comparison of metabolic capacities of organisms at genome level. *Mol Phylogenet Evol*, **31**, 204-213.
11. Huson, D.H. and Steel, M. (2004) Phylogenetic trees based on gene content. *Bioinformatics*, **20**, 2044-2049.
12. Saitou, N. and Nei, M. (1987) The neighbor-joining method: a new method for reconstructing phylogenetic trees. *Mol Biol Evol*, **4**, 406-425.
13. Fitz-Gibbon, S.T. and House, C.H. (1999) Whole genome-based phylogenetic analysis of free-living microorganisms. *Nucleic Acids Res*, **27**, 4218-4222.
14. Tekaia, F., Lazcano, A. and Dujon, B. (1999) The genomic tree as revealed from whole proteome comparisons. *Genome Res*, **9**, 550-557.
15. Lin, J. and Gerstein, M. (2000) Whole-genome trees based on the occurrence of folds and orthologs: implications for comparing genomes on different levels. *Genome Res*, **10**, 808-818.
16. House, C.H. and Fitz-Gibbon, S.T. (2002) Using homolog groups to create a whole-genomic tree of free-living organisms: an update. *J Mol Evol*, **54**, 539-547.
17. Korbel, J.O., Snel, B., Huynen, M.A. and Bork, P. (2002) SHOT: a web server for the construction of genome phylogenies. *Trends Genet*, **18**, 158-162.
18. Lake, J.A. and Rivera, M.C. (2004) Deriving the genomic tree of life in the presence of horizontal gene transfer: conditioned reconstruction. *Mol Biol Evol*, **21**, 681-690.
19. Rivera, M.C. and Lake, J.A. (2004) The ring of life provides evidence for a genome fusion origin of eukaryotes. *Nature*, **431**, 152-155.
20. Forst, C.V. and Schulten, K. (1999) Evolution of metabolisms: a new method for the

comparison of metabolic pathways using genomics information. *J Comput Biol*, **6**, 343-360.

21. Hong, S.H., Kim, T.Y. and Lee, S.Y. (2004) Phylogenetic analysis based on genome-scale metabolic pathway reaction content. *Appl Microbiol Biotechnol*, **65**, 203-210.
22. Forst, C.V., Flamm, C., Hofacker, I.L. and Stadler, P.F. (2006) Algebraic comparison of metabolic networks, phylogenetic inference, and metabolic innovation. *BMC Bioinformatics*, **7**, 67.
23. Bowers, P.M., Pellegrini, M., Thompson, M.J., Fierro, J., Yeates, T.O. and Eisenberg, D. (2004) Prolinks: a database of protein functional linkages derived from coevolution. *Genome Biol*, **5**, R35.
24. Podani, J., Oltvai, Z.N., Jeong, H., Tombor, B., Barabasi, A.L. and Szathmary, E. (2001) Comparable system-level organization of Archaea and Eukaryotes. *Nat Genet*, 29, 54-56.
